# Tumor-associated antigen PRAME exhibits dualistic functions that are targetable in diffuse large B cell lymphoma

**DOI:** 10.1172/JCI145343

**Published:** 2022-05-16

**Authors:** Katsuyoshi Takata, Lauren C. Chong, Daisuke Ennishi, Tomohiro Aoki, Michael Yu Li, Avinash Thakur, Shannon Healy, Elena Viganò, Tao Dao, Daniel Kwon, Gerben Duns, Julie S. Nielsen, Susana Ben-Neriah, Ethan Tse, Stacy S. Hung, Merrill Boyle, Sung Soo Mun, Christopher M. Bourne, Bruce Woolcock, Adèle Telenius, Makoto Kishida, Shinya Rai, Allen W. Zhang, Ali Bashashati, Saeed Saberi, Gianluca D’Antonio, Brad H. Nelson, Sohrab P. Shah, Pamela A. Hoodless, Ari M. Melnick, Randy D. Gascoyne, Joseph M. Connors, David A. Scheinberg, Wendy Béguelin, David W. Scott, Christian Steidl

**Affiliations:** 1 Centre for Lymphoid Cancer, British Columbia Cancer, Vancouver, British Columbia, Canada.; 2 Division of Molecular and Cellular Pathology, Niigata University Graduate School of Medical and Dental Sciences, Niigata, Japan.; 3 Department of Medical Genetics, University of British Columbia, Vancouver, British Columbia, Canada.; 4 Terry Fox Laboratory, British Columbia Cancer, Vancouver, British Columbia, Canada.; 5 Molecular Pharmacology Program, Memorial Sloan Kettering Cancer Center, New York, New York, USA.; 6 Department of Molecular Oncology and; 7 Trev and Joyce Deeley Research Centre, British Columbia Cancer, Vancouver, British Columbia, Canada.; 8 Department of Epidemiology & Biostatistics, Memorial Sloan Kettering Cancer Center, New York, New York, USA.; 9 Division of Hematology and Medical Oncology, Department of Medicine, Weill Cornell Medical College, New York, New York, USA.; 10 Department of Pathology and Laboratory Medicine, University of British Columbia, Vancouver, British Columbia, Canada.

**Keywords:** Hematology, Oncology, Lymphomas

## Abstract

PRAME is a prominent member of the cancer testis antigen family of proteins, which triggers autologous T cell–mediated immune responses. Integrative genomic analysis in diffuse large B cell lymphoma (DLBCL) uncovered recurrent and highly focal deletions of 22q11.22, including the *PRAME* gene, which were associated with poor outcome. *PRAME*-deleted tumors showed cytotoxic T cell immune escape and were associated with cold tumor microenvironments. In addition, PRAME downmodulation was strongly associated with somatic *EZH2* Y641 mutations in DLBCL. In turn, PRC2-regulated genes were repressed in isogenic *PRAME*-KO lymphoma cell lines, and PRAME was found to directly interact with EZH2 as a negative regulator. EZH2 inhibition with EPZ-6438 abrogated these extrinsic and intrinsic effects, leading to PRAME expression and microenvironment restoration in vivo. Our data highlight multiple functions of PRAME during lymphomagenesis and provide a preclinical rationale for synergistic therapies combining epigenetic reprogramming with PRAME-targeted therapies.

## Graphical abstract

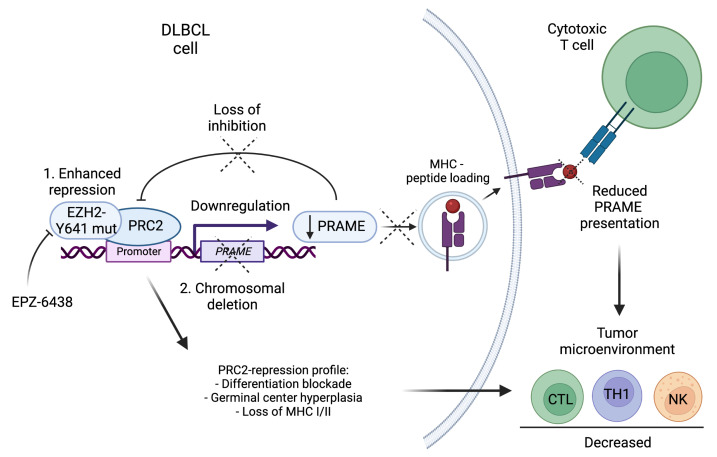

## Introduction

The current treatment standard of combined immunochemotherapy with rituximab, cyclophosphamide, doxorubicin, vincristine, and prednisone (R-CHOP) has achieved significant improvement in patient outcomes in diffuse large B cell lymphoma (DLBCL) over the past 15 years (1–3). However, approximately 40% of patients with DLBCL experience relapse or refractory disease. Therefore, the development of new therapeutic strategies for treatment-resistant disease is an urgent unmet clinical need in DLBCL. With the goal to translate biological discovery into clinical actionability, 2 major research foci have emerged: a) characterization of tumor cell genetics and associated cell-autonomous phenotypes and b) explication of crosstalk in the cellular ecosystem of the tumor microenvironment (TME).

Several genetic landscape studies using next-generation sequencing have contributed to a near-complete description of the most prevalent somatic gene alterations and structural genomic changes in the context of transcriptionally defined DLBCL subtypes, such as the cell-of-origin classification (4–7). However, comparatively little is known about the immune biology of DLBCL as reflected in clonal selection of specific somatic gene mutations in response to immune system pressure and the specific composition of the TME. The TME of DLBCL mainly consists of nonmalignant immune cells, such as T cells, NK cells, macrophages, and stromal cells. In recent studies, the TME has been shown to play a key role in tumor cell maintenance, immune escape, and treatment failure (8, 9). Given the importance of the activation of immune effector cells to eliminate cancer, a number of immunotherapies, such as programmed cell death 1 (PD-1) blockade, chimeric antigen receptor (CAR) T cells, and T cell–engaging antibodies, have been evaluated and been FDA approved in several types of cancer, including lymphoma (10–13). However, deeper insight into immune biology that can lead to the development of more specific therapeutics and can guide rational development and selection across an increasing number of available immunotherapies is still lacking.

In the context of developing new immunotherapies, targeting tumor-associated antigens (TAAs) that are presented by MHC on tumor cells is a promising therapeutic strategy for patients who experience treatment failure (14, 15). TAAs are presented by MHC class I and II molecules and involve recognition by TAA-specific CD8^+^ and CD4^+^ T cells, both of which may be required for tumor elimination (16, 17). Hence, expression of both MHC I and II on tumor cells may be essential for productive immune responses to epitopes. A recently published study from our group established the importance of MHC class I and II expression for reduction of immunogenicity, with implications for treatment outcome, TME composition, and therapeutic considerations pertaining to epigenetic reprogramming and combination immunotherapy (18). However, it remains unclear in the current literature whether MHC-dependent presentation of specific TAAs and additional somatically acquired immune escape mechanisms play a role in the pathogenesis of DLBCL.

To address this open question, we performed an integrative genomic analysis of whole-transcriptome RNA-Seq, targeted genomic sequencing, and high-resolution copy number (CN) analysis in a previously reported cohort of 347 DLBCL tumors from patients uniformly treated with R-CHOP (18, 19). We discovered recurrent and highly focal deletions of 22q11.22, including the *PRAME* gene (44/338, 13%), as a clinically and genetically relevant CN alteration in germinal center B cell–like (GCB) type DLBCL. PRAME is a prominent member of the cancer testis antigen family of proteins that is expressed in various types of cancers but generally not in normal tissues apart from male germinal cells (20, 21). PRAME is highlighted as a new cancer therapeutic target of T cell– or antibody-based immunotherapies, with promising antitumor responses in early-phase clinical trials and preclinical models for several types of cancers (22–26). In our study, PRAME loss was linked to an immunologically “cold” TME in GCB-DLBCL, and PRAME loss demonstrated immune evasion from cytotoxic T cells in vitro. Furthermore, enhancer of zeste homolog 2–activating (EZH2-activating) mutations suppressed PRAME expression. Conversely, PRAME was found to show repressive activity on EZH2 activity, notably through direct interaction. EZH2 is the catalytic component of the polycomb repressive complex 2 (PRC2), which is responsible for H3K27 methylation activity (mono-, di-, trimethyl) and has a key role for cell development and differentiation (27). *EZH2* mutations, especially hotspot mutations affecting Y641, drive B cell lymphoma development, and EZH2 inhibition is considered an attractive therapeutic option in *EZH2*-mutated B cell lymphomas, including DLBCL (28, 29). Our data elucidated dualistic functions of PRAME, including cell-autonomous regulation (“intrinsic”) and TME biology (“extrinsic”) implications as a consequence of frequently observed PRAME loss or downmodulation. Furthermore, these dual effects can be dynamically altered through PRAME restoration using an EZH2 inhibitor (EZH2i), providing a preclinical rationale for a 2-pronged attack of cancer cells.

## Results

### PRAME deletions are associated with GCB-DLBCL and poor treatment outcome.

**Figure 1:**
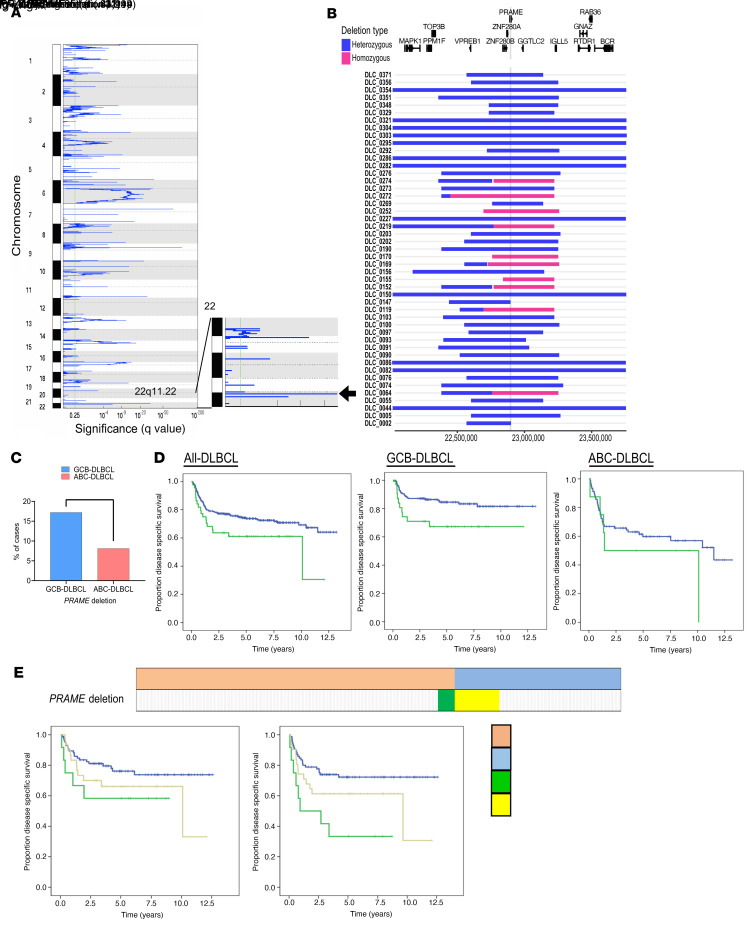
Discovery and clinical impact of *PRAME* deletion in DLBCL. (**A**) Significant focal deletions identified by GISTIC 2.0 analysis (*n* = 338) and highlighted focal deletion in 22q11.22 region (arrow). (**B**) Location of focal deletions in the 22q11.22 locus are shown with rectangles. Blue shows heterozygous deletion and red shows homozygous deletions. The location of genes in this region is represented above. (**C**) Frequency of *PRAME* deletion between GCB-DLBCL and ABC-DLBCL. A χ^2^ test was used to compare these frequencies (**P < 0.01). (**D**) Kaplan-Meier curves represent disease-specific survival according to *PRAME* deletion status among all-DLBCL, GCB-DLBCL, and ABC-DLBCL. (**E**) Correlation among Ig-κ, Ig-λ rearrangement, and *PRAME* deletion status in DLBCL. Cases in green and yellow correspond to Ig-κ rearrangement with *PRAME* deletion, and cases in light red and light blue correspond to Ig-λ rearrangement with *PRAME* deletion. Kaplan-Meier curves represent disease-specific survival (left) and time to progression (right) according to Ig-κ, Ig-λ rearrangement, and *PRAME* deletion status.

We performed CN analysis using high-resolution SNP6.0 arrays (Figure 1A) in a previously published de novo DLBCL cohort of 347 tumors from patients (19). Several alterations in previously reported gene loci, including amplifications (*NFKBIZ*, *BCL6*, *CD274*, *NFATC1*, and *BCL2*) and deletions (*IKBKE*, *ITPKB*, *PRDM1*, and *CDKN2A*), were found (4, 30). Notably, we discovered recurrent and highly focal deletions of 22q11.22, including the *PRAME* gene, in 13% (44/338) of the cases. Of these, 10 cases were predicted to harbor homozygous *PRAME* deletions. These deletions were clustered in a narrow chromosomal region that included a very small number of genes (*VpreB1*, *ZNF280A/B*, *PRAME*, *GGTLC2*, and *miR-650*) (Figure 1B) and were located close to the Ig-λ gene. Deletions of 22q11.22 (*PRAME* deletions) were found significantly enriched in GCB type DLBCL (GCB type: 17% [31/180] vs. activated B cell (ABC) type: 8% [8/98], *P* < 0.01; Figure 1C). *PRAME* deletions were significantly associated with poor treatment outcome in GCB-DLBCL. The 5-year disease-specific survival (DSS) was 84.5% in *PRAME*-CN-neutral cases versus 67.2% in *PRAME*-deleted cases (*P* = 0.03) (Figure 1D). The 5-year time to progression (TTP) was 79.3% in *PRAME*-CN-neutral cases versus 57.7% in *PRAME*-deleted cases (*P* = 0.009) (Supplemental Figure 1; supplemental material available online with this article; https://doi.org/10.1172/JCI145343DS1). Using an independent de novo DLBCL cohort uniformly treated with R-CHOP (*n* = 52; ref. 31), we confirmed that *PRAME* deletions were correlated with inferior treatment outcome (3-year DSS: *PRAME* CN neutral: 77.5% vs. *PRAME* deleted: 42.9%, *P* = 0.021, 3-year TTP: *PRAME* CN neutral: 77.5% vs. *PRAME* deleted: 42.9%, *P* = 0.021, Supplemental Figure 1). Clinical and pathological characteristics of patients with *PRAME*-CN-neutral and *PRAME*-deleted tumor cells are shown in Supplemental Tables 1 and 2. Among 347 DLBCL cases, no significant differences between patients with *PRAME* CN neutral and deleted tumor cells were observed for age, sex, stage, or International Prognostic Index score, though *PRAME*-deleted samples were significantly enriched in Ig-λ–rearranged tumors. Ig-λ–rearranged tumors were similarly trending toward poor prognosis in GCB-DLBCL (*P* = 0.096; Supplemental Figure 2A). When studying *PRAME*-deleted/Ig-λ–rearranged tumors, we found that *PRAME* deletion was prognostically more significant than Ig-λ rearrangement. The 5-year DSS was 76.2% in Ig-λ rearrangement only versus 64.0% in Ig-λ rearrangement plus *PRAME* deletion versus 58.3% in Ig-κ rearrangement plus *PRAME* deletion. The 5-year TTP was 72.2% in Ig-λ rearrangement only versus 61.3% in Ig-λ rearrangement plus *PRAME*-deletion versus 33.3% in Ig-κ rearrangement plus *PRAME* deletion (Figure 1E). Moreover, in a pairwise multivariable analysis, *PRAME* deletion remained an independent prognostic biomarker for both DSS and TTP (*P* = 0.050, HR = 1.67 [95% CI: 1.00–2.78], *P* = 0.03, HR = 1.68 [95% CI: 1.06–2.67]).

We looked in more detail into the 5 genes, including *PRAME*, which were involved in the minimally deleted region on chromosome 22q11.22, and correlated each mRNA level with deletion status. We found that only *PRAME* and *ZNF280B* mRNA levels were significantly correlated with 22q11.22 deletion status, whereas the other 3 genes were not significant (Supplemental Figure 2B). We also correlated treatment outcomes with mRNA status and found that the *PRAME*-low mRNA group had significantly shorter DSS than the *PRAME*-high group when setting the quartile cutoff value at 25%, whereas the other 4 genes did not show any significance (Supplemental Figure 2C). In aggregate, we prioritized *PRAME* as the best candidate gene within the 22q11.22 deleted region for follow-up studies.

IHC using PRAME antibodies showed 104/347 (30%) cases were positive (score +1 or +2) for PRAME protein expression, and results were concordant between PRAME expression and CN deletion status (Supplemental Figure 3, A and B). Although statistical significance was not reached, PRAME IHC–negative patients showed a trend toward worse treatment outcomes in GCB-DLBCL (Supplemental Figure 4). Previous literature describes HLA-A*0201–specific presentation of PRAME peptides (32), so we next determined HLA haplotypes in the *PRAME*-deleted cases. Of note, patients with *PRAME*-deleted tumors were more frequently of the HLA-A*0201 haplotype than patients with *PRAME*-CN-neutral tumors (*P* = 0.005; Supplemental Table 1). In aggregate, these data indicate that *PRAME* deletions are a genetically and clinically relevant CN alteration in GCB-DLBCL, which motivated us to perform further phenotypic and functional studies to elucidate potential oncogenic driver capacities.

### PRAME deletions are associated with an immunologically cold TME.

**Figure 2:**
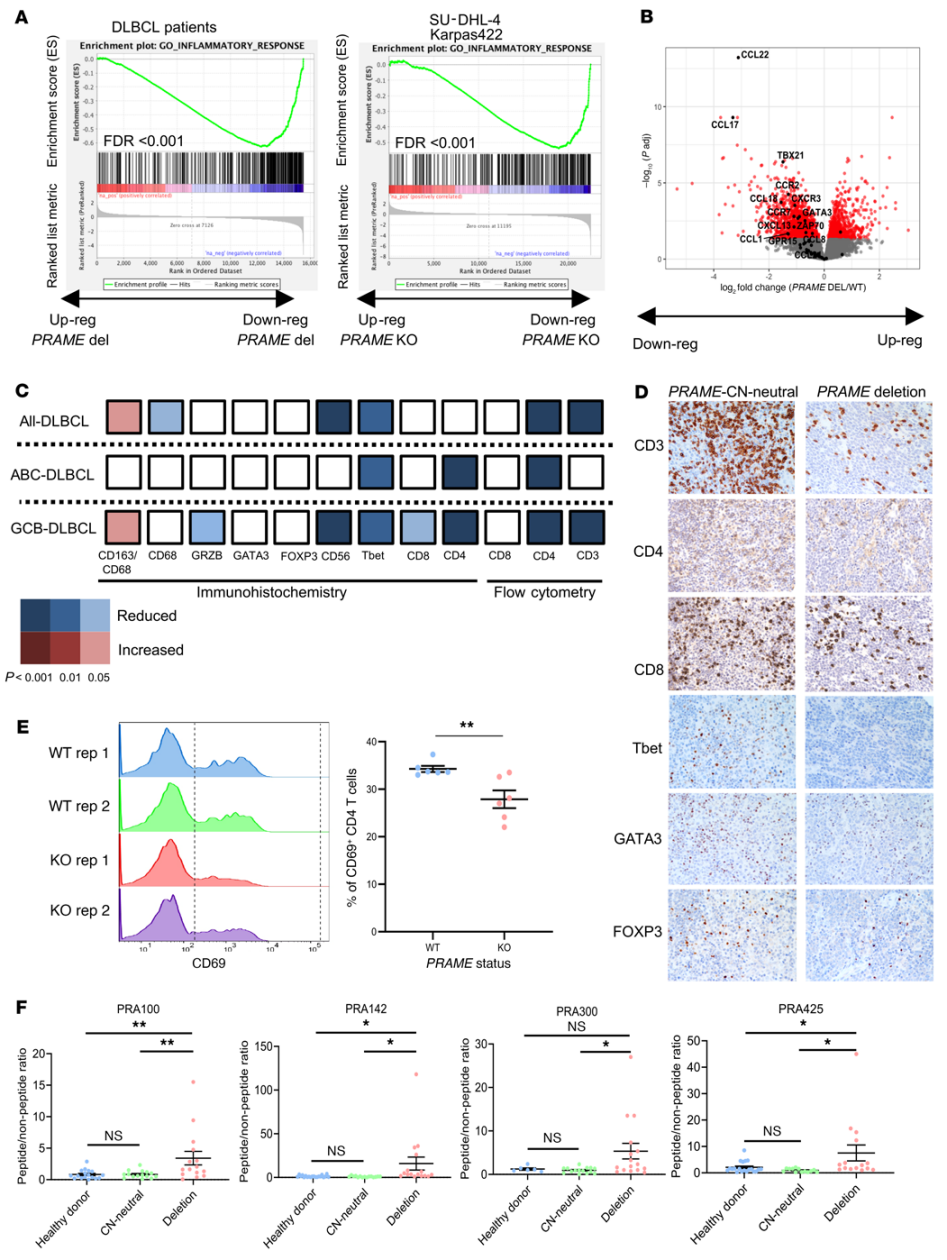
PRAME deletion correlates with immunologically cold TME status. (**A**) Preranked GSEA enrichment plots of inflammatory response genes in *PRAME*-CN-neutral versus *PRAME*-deleted patient samples (left) and in *PRAME-*WT versus *PRAME*-KO cell lines (right). (**B**) Volcano plot of downregulated and upregulated genes in *PRAME*-deleted versus *PRAME*-CN-neutral patient samples. Red dots show genes that are significantly differentially expressed (adjusted *P* < 0.05), and genes involved in lymphocyte migration are highlighted in black. (**C**) Validation of TME components using flow cytometry and IHC data in *PRAME*-CN-neutral versus *PRAME*-deleted patient samples. (**D**) Representative IHC figures between *PRAME*-CN-neutral versus *PRAME*-deleted patient samples. (**E**) CD4^+^ naive T cell coculture with Karpas-422 PRAME isogenic cell line system (left: histogram, right: summary of CD69^+^CD4^+^ T cell population). Unpaired 2-tailed *t* test, mean ± SEM, **P < 0.01. (**F**) Summary of IFN-γ ELISPOT data of healthy donor, *PRAME*-CN-neutral, and *PRAME*-deleted patient derived T cells using 4 peptides (peptide/nonpeptide ratio) (Bonferroni’s multiple-comparison test, mean ± SEM, *P < 0.05, **P < 0.01).

To explore *PRAME* deletion–associated phenotypes, we next analyzed the corresponding RNA-Seq data from a subset of the de novo DLBCL cohort (*n* = 311), comparing expression profiles of the *PRAME*-CN-neutral and *PRAME*-deleted samples. *PRAME* deletion was significantly correlated with *PRAME* mRNA expression (Supplemental Figure 3B). Downregulation of the inflammatory response and immune response pathways were listed in the top 10 pathways associated with *PRAME* deletion (Supplemental Tables 3 and 4). Moreover, preranked gene set enrichment analysis (GSEA) using log_2_ fold change revealed that inflammatory response and lymphocyte migration signatures were downregulated in *PRAME*-deleted tumors (Figure 2A). We generated isogenic *PRAME*-KO cell line systems using CRISPR/Cas9 genome editing in GCB-DLBCL cell lines SU-DHL-4 and Karpas-422 and ABC-DLBCL cell line HBL-1. Effective genome editing was confirmed by Sanger sequencing and Western blotting (Supplemental Figure 5, A and B). We performed RNA-Seq analysis on these cell lines, with and without genome editing, to derive *PRAME*-KO–associated gene signatures. GSEA confirmed the downregulation of inflammatory response signature genes in SU-DHL-4 and Karpas-422 with *PRAME*-KO cells as observed in primary samples (Figure 2A). Of note, mRNA levels of several cytokines, chemokines, and chemokine ligands, such as CCL22, CCL17, CCL18, and CXCL13, were reduced in *PRAME*-deleted patient samples, and TLR7, CCL3, CCL22, and CXCL11 were reduced in *PRAME*-KO cell lines (Figure 2B and Supplemental Tables 5 and 6). Analysis by flow cytometry and IHC data of the primary DLBCL samples further revealed that T cells and NK cells (CD3, CD4 by flow cytometry; CD4, CD8, T-bet, CD56, granzyme B by IHC) were significantly reduced in *PRAME*-deleted patient samples (Figure 2, C and D), especially in GCB-DLBCL. In aggregate, these results suggest that *PRAME*-deleted tumors are characterized by an immunologically cold TME.

### In vitro coculture model of proposed immunologically cold TME.

To demonstrate a direct link of PRAME loss with phenotypic changes in the TME, we studied T cell populations cocultured with HBL-1 (*EZH2*-WT), Karpas-422, and SU-DHL-4 (both EZH2 mutated, *EZH2*-mut) *PRAME*–isogenic KO cell line systems, using MHC I/II blockade conditions because donor PBMCs were not HLA matched. CD69 positivity and T cell proliferation were compared in cocultures with WT versus PRAME-KO cells. In HBL-1, the CD69^+^ T cell population was significantly reduced when cocultured with *PRAME*-KO cells as observed using flow cytometry (*P* = 0.0006). T cell proliferation assessed by CellTrace violet dye was also significantly reduced in the *PRAME*-KO group (*P* = 0.0067; Supplemental Figure 6). In Karpas-422, the CD8^+^CD69^+^ population was not significantly different between *PRAME*-WT and -KO groups, although we observed a significant difference in the activation status of cocultured naive CD4^+^ T cells (CD4^+^CD69^+^ population; Figure 2E and Supplemental Figure 6B), matching the findings in the primary DLBCL samples (Figure 2C). No activation differences in either the CD4^+^ and CD8^+^ cells were found in SU-DHL-4 coculture models (Supplemental Figure 6, C and D).

### PRAME deletions represent an immune evasion mechanism.

The RNA-Seq, IHC, and in vitro coculture experiment data from our de novo DLBCL cohort suggested *PRAME* deletion as a mechanism of immune escape through reduction of immunogenicity. To investigate whether patient-derived T cells respond to PRAME antigen, we performed IFN-γ enzyme-linked immunospot (ELISPOT) assays with PBMC samples from 4 patients with *PRAME*-CN-neutral (2 of 4 HLA-A2^+^) tumors, 4 patients with *PRAME*-deleted tumors (2 of 4 HLA-A2^+^), and 6 healthy donors. We independently tested 4 PRAME peptides (PRA 100, 142, 300, and 425) using previously published peptide sequences (32). T cells from patients with *PRAME*-deleted tumors showed significantly increased reactivity to PRAME peptides. By contrast, T cells from patients with CN-neutral tumors or healthy donors showed no reactivity (Figure 2F and Supplemental Figure 7).

### Somatic EZH2 mutations and PRAME deletions converge to downmodulate PRAME expression.

**Figure 3:**
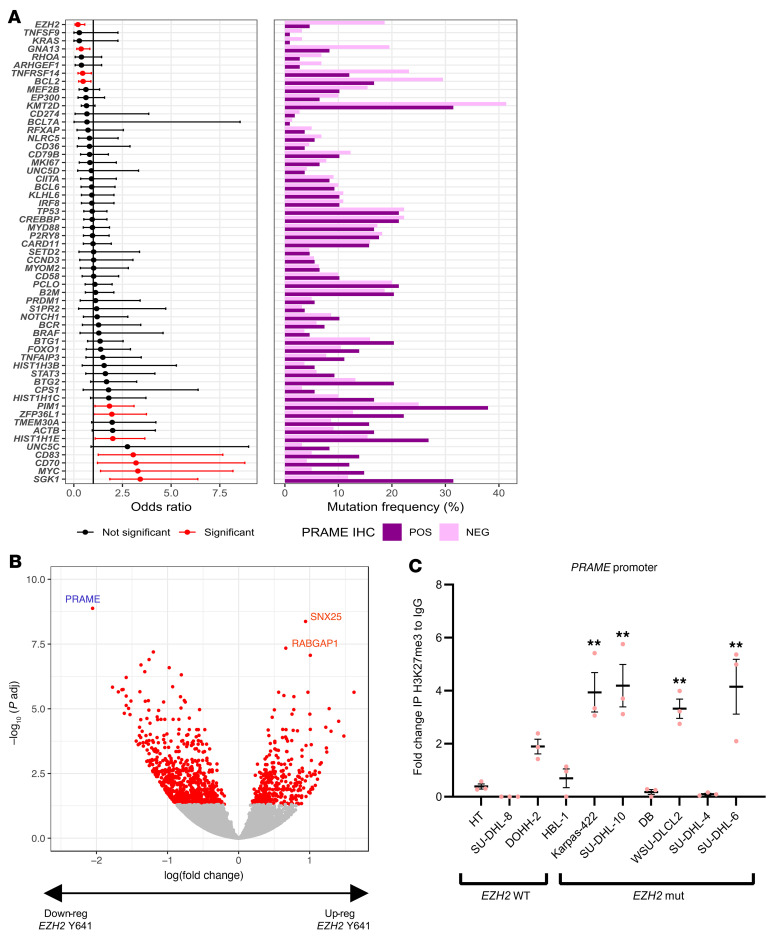
*EZH2* Y641 hotspot mutations are significantly enriched in PRAME-negative cases and functionally suppress PRAME expression via promoter binding. (**A**) Forest plot shows association with somatic status between PRAME IHC-positive and IHC-negative samples. Red bars indicate statistical significance. The frequency of gene mutations is shown on the right and based on PRAME IHC status. (**B**) Volcano plot of downregulated and upregulated genes in *EZH2*-mutated versus WT samples (adjusted *P* < 0.05). (**C**) H3K27me3 ChIP quantitative PCR analysis on PRAME promoter region between *EZH2*-WT cell lines (HT, SU-DHL-8, DOHH-2, and HBL-1) and mutated cell lines (Karpas-422, SU-DHL-10, DB, WSU-DLCL2, SU-DHL-4, and SU-DHL-6). Data were normalized by *ACTB* (Bonferroni’s multiple-comparison test, mean ± SEM, **P < 0.01).

To more comprehensively characterize genetic events underlying PRAME downmodulation, we next investigated associations of somatic genetic alterations with PRAME protein expression (IHC positive: 30%, IHC negative: 70%) in our DLBCL cohort. Using deep, targeted amplicon sequencing of the most common gene mutations found in DLBCL (*n* = 57 genes; Supplemental Figure 8), we observed recurrent mutations, including *SGK1* (*P* < 0.001), *MYC*, *CD83*, *CD70* (*P* < 0.01), *PIM1*, *HIST1H1E*, and *ZFP36L1* (*P* < 0.05) in PRAME-positive cases. In addition, we identified that *EZH2* mutations were significantly less frequent in PRAME-positive cases (*P* = 0.00033) (Figure 3A). Importantly, PRAME expression showed a strong negative correlation with the presence of an *EZH2* Y641 hotspot mutation, and *PRAME* was the top-listed downregulated gene in cases with an *EZH2* hotspot mutation (Figure 3B and Supplemental Table 7).

**Figure 4:**
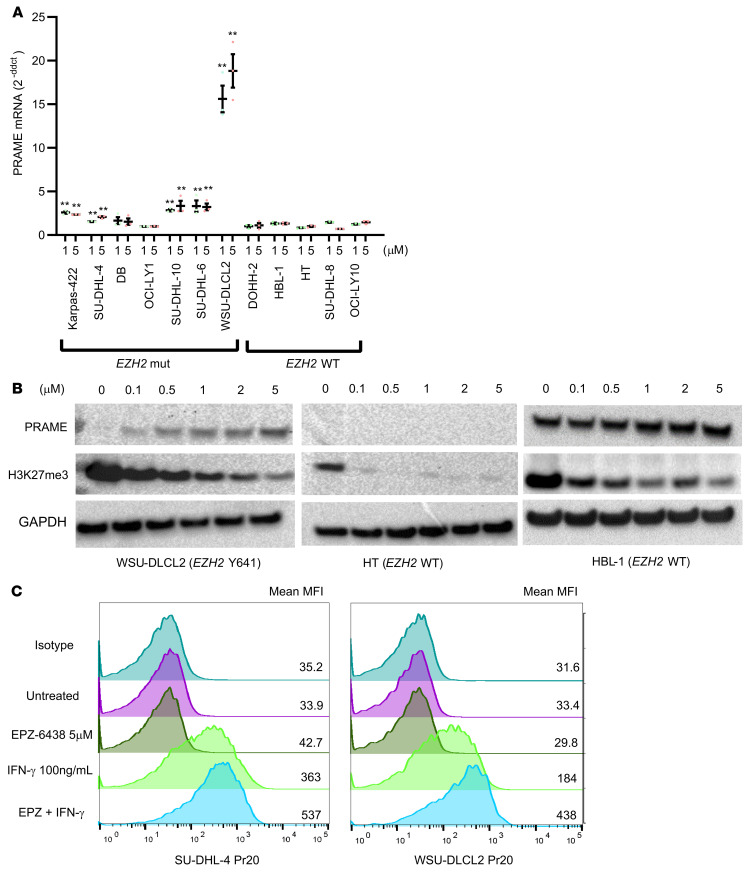
PRAME restoration and Pr20 antibody binding enhancement by EZH2 inhibition. (**A**) PRAME real-time PCR of EPZ-6438–treated cell lines in 3 concentrations (0, 1, 5 μM) (Bonferroni’s multiple-comparison test, mean ± SEM, **P < 0.01). Data were normalized to expression values of the no-treatment control (0 μM). (**B**) Representative immunoblotting of EPZ-6438–treated cell lysates in 6 concentrations (0, 0.1, 0.5, 1, 2, 5 μM) for WSU-DLCL2 (*EZH2* mut) and HT, HBL-1 (*EZH2* WT) cell lines. EZH2 inhibition was evaluated by H3K27me3 immunoblotting. (**C**) Pr20 antibody binding enhancement using EPZ-6438, IFN-γ, and the combination of EPZ-6438 and IFN-γ.

Given the correlation of *EZH2* Y641 hotspot mutations with PRAME protein expression and CN status (Supplemental Figure 9), we hypothesized that *EZH2* mutations might directly regulate *PRAME* gene expression. To examine the mechanism of *PRAME* regulation by EZH2, we performed ChIP assays in *EZH*2-WT (DOHH2, HT, HBL-1, and SU-DHL-8) and *EZH2*-mut DLBCL cell lines (Karpas-422, SU-DHL-10, DB, WSU-DLCL2, SU-DHL-4, and SU-DHL-6) using anti-H3K27me3 antibody. ChIP and qPCR analysis revealed that the amount of H3K27me3 in the PRAME promoter region was higher in *EZH*2-mut cell lines than in *EZH2*-WT cell lines (Figure 3C). Based on these results, we examined whether PRAME expression would be restored using the EZH2i EPZ-6438 in 12 DLBCL cell lines (7 *EZH2* mut, 5 WT). Upon application of EPZ-6438, 5 of 7 *EZH2*-mut cell lines showed significant PRAME restoration (1.5–20 times PRAME mRNA increase compared with vehicle), whereas all 5 WT cell lines did not show any significant PRAME restoration (Figure 4, A and B, and Supplemental Figure 10). Using EZH2 knockdown by RNA interference, we also confirmed PRAME restoration in WSU-DLCL2 cells, suggesting direct regulation by *EZH2* (Supplemental Figure 11).

To further explore the enhancement of MHC-dependent PRAME peptide presentation upon EZH2 inhibition, we performed a PRAME antibody binding assay using a previously established T cell receptor mimic PRAME antibody (Pr20; ref. 22). Among 5 HLA-A2^+^ DLBCL cell lines, Pr20 binding was enhanced only in the Karpas-422 cell line by EPZ-6438 treatment but not in other cell lines (SU-DHL-4, WSU-DLCL2, and DB, Supplemental Figure 12). To support PRAME peptide presentation by MHC I molecules in our in vitro system, we performed the Pr20 binding assay under IFN-γ stimulation, which enhances immunoproteasome activation. The combination of IFN-γ and EPZ-6438 treatment enhanced Pr20 binding in 4 of 5 cell lines (Karpas-422, RL, WSU-DLCL2, SU-DHL-4; Figure 4C). These results suggest that restoration of PRAME antigen presentation by EZH2 inhibition could enhance PRAME-dependent immunotherapy responses in DLBCL.

### EZH2 inhibition induces PRAME expression and immune infiltrates in Ezh2-mutant lymphomas in vivo.

The association of EZH2 with the PRAME promoter and the transcriptional repression of PRAME in *EZH2*-mut lymphomas prompted us to explore whether EZH2-targeted therapy might induce PRAME and reactivate antitumor immunity in vivo. To address this question, we developed a syngeneic animal model for *Ezh2*-mut lymphoma that was suitable for preclinical therapeutic studies. We previously reported that mice engineered for conditional expression of *Ezh2^Y641F^* and *Bcl6* in GCB cells (IμBcl6 *Ezh2^Y641F^* Cγ1-Cre) develop GC-derived DLBCLs similar to the human disease with high penetrance (33). Generating these mice through breeding is time-consuming and tumor onset is heterogeneous. In order to adopt this model for use in preclinical studies, we transplanted IμBcl6 *Ezh2^Y641F^* Cγ1-Cre into lethally irradiated recipient mice. Once engrafted, mice were immunized every 3 weeks to ensure continuous formation of GCs, which is required for activation of the 2 oncogenes. Mice were euthanized upon onset of splenomegaly as a surrogate for development of lymphoma, which we confirmed by histologic analysis of spleens and other tissues (data not shown).

**Figure 5:**
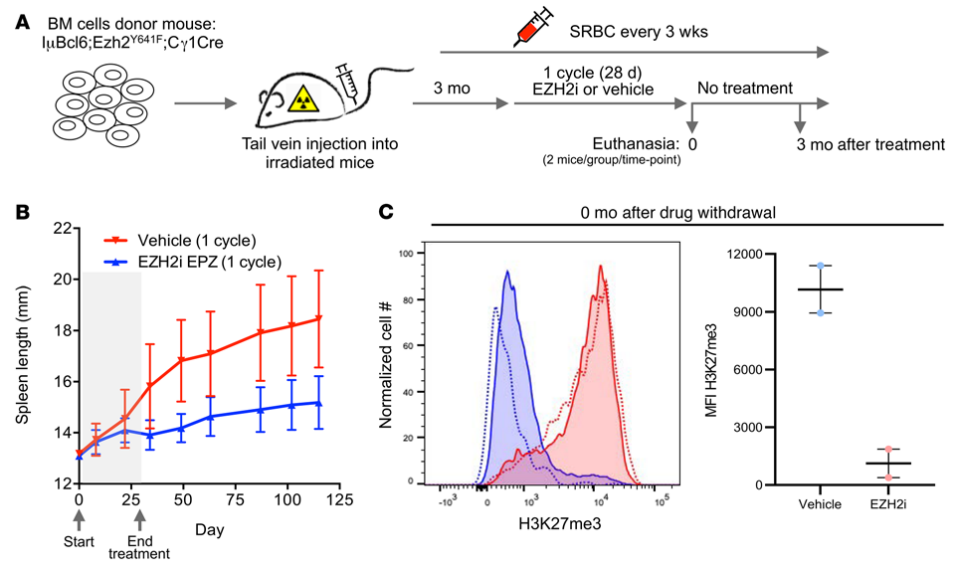
EZH2 inhibition in an *Ezh2*-mutant in vivo lymphoma model. (**A**) Generation of IμBcl*6 Ezh2Y^641F^* Cγ1-Cre mice and EZH2 inhibitor treatment. (**B**) Spleen length change after treatment with vehicle or EZH2 inhibitor. (**C**) H3K27me3 expression in splenocytes immediately after EZH2 inhibitor treatment. Representative histogram (left) and MFI (right).

**Figure 6:**
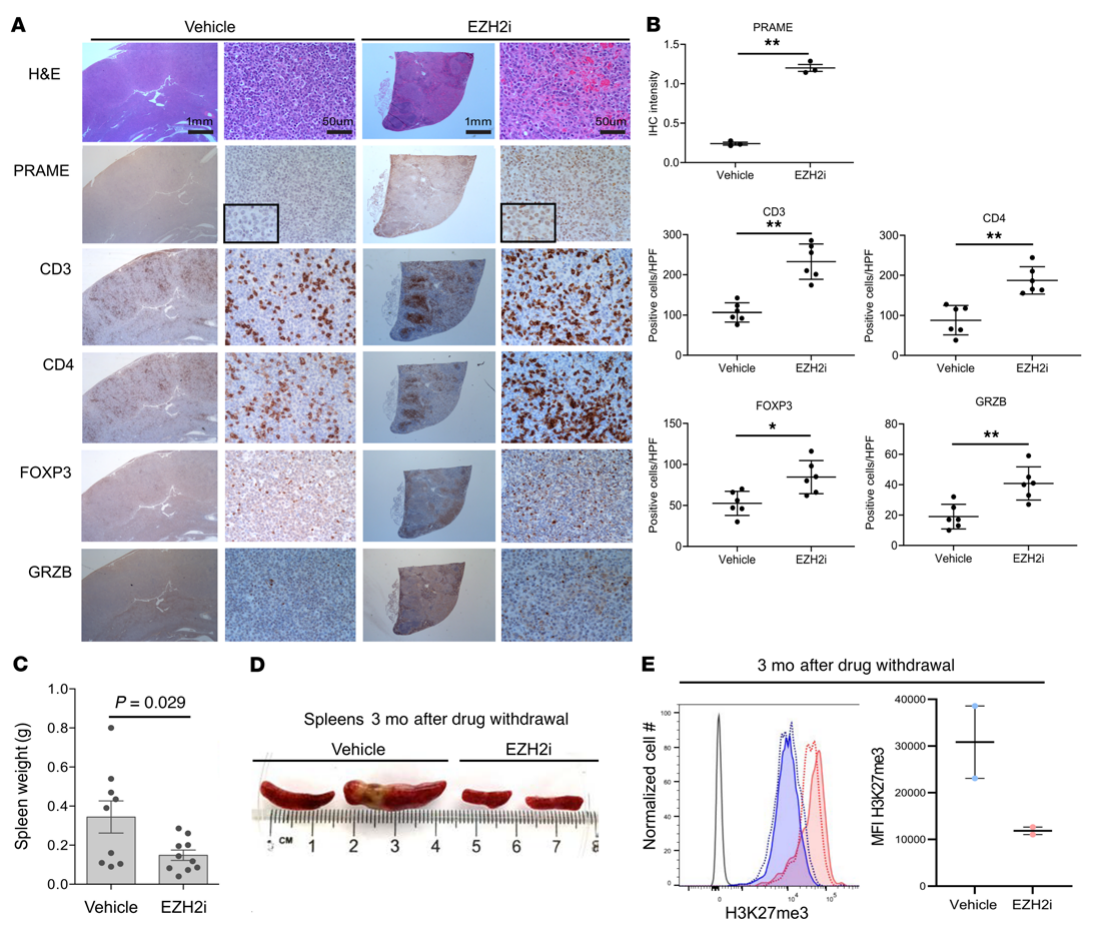
PRAME restoration and immune infiltrates change by EZH2 inhibitor in an *Ezh2*-mutant in vivo lymphoma model. (**A**) Serially sectioned tissues were stained with H&E, PRAME, CD3, CD4, FOXP3, and granzyme B (×20 and ×400 original magnification). Scale bar: 1 mm. (**B**) Comparison of intensity for PRAME and proportions of CD3-, CD4-, FOXP3-, and granzyme B–positive cells between vehicle and EPZ011989 treatment. Positive cells were counted in 3 independent high-power fields (*P < 0.05, **P < 0.01). (**C**) Spleen weight comparison between vehicle and EZH2 inhibitor treatment group. (**D**) Representative images of spleens from mice euthanized after 3 months of treatment. (**E**) H3K27me3 expression of splenocytes 3 months after treatment with vehicle or EZH2 inhibitor. Representative histogram (left) and MFI (right).

Based on this experience, we generated a cohort of 30 IμBcl6 *Ezh2^Y641F^* Cγ1-Cre mice and observed onset of splenomegaly occurring at 3 months after transplant. Upon onset of splenomegaly, mice were randomized to treatment with 250 mg/kg EZH2i EPZ011989 or vehicle for 28 consecutive days (Figure 5A). During this time, most of the mice treated with vehicle developed progressive splenomegaly, whereas those treated with EZH2i maintained stable disease (Figure 5B). Two mice per group were euthanized the day after completion of treatment for histologic analysis. We measured the abundance of H3K27me3 in splenocytes from these animals and observed near total loss of this mark in EZH2i-treated mice (Figure 5C), confirming the in vivo activity of the compound. We then examined the spleens of these animals by IHC and observed that EZH2i induced PRAME expression in the treated lymphomas as compared with controls (*P* < 0.001; Figure 6, A and B). IHC for CD3, CD4, FOXP3, and granzyme B revealed significant increases in these various T cell populations after EZH2i treatment (Figure 6, A and B). In conjunction with our findings in the in vitro coculture models, these data suggest that EZH2i alters TME biology at least in part via PRAME restoration and a related feedback loop to the PCR2 complex with downstream gene expression profile changes.

To determine whether this immune reactivation is associated with longer-term suppression of these lymphomas, we followed the remaining mice (13 per group) for an additional 3 months without further treatment. Strikingly, we observed that EZH2i resulted in sustained suppression of lymphoma throughout the 3-month treatment-free window, manifesting as reduced spleen weights and size (Figure 5B and Figure 6, C and D). Notably, we also observed sustained reduction of H3K27me3 in splenocytes from these animals (Figure 6E), suggesting that loss of this histone mark was epigenetically sustained for a long time after treatment. This long-term effect of EZH2i is consistent with the immune reactivation observed after completion of the treatment cycle and highlights the potential significance of upregulation of PRAME and other immune modulatory genes that are silenced by mutant EZH2 in DLBCL cells.

### PRAME directly interacts with EZH2 and influences H3K27me3 levels.

**Figure 7:**
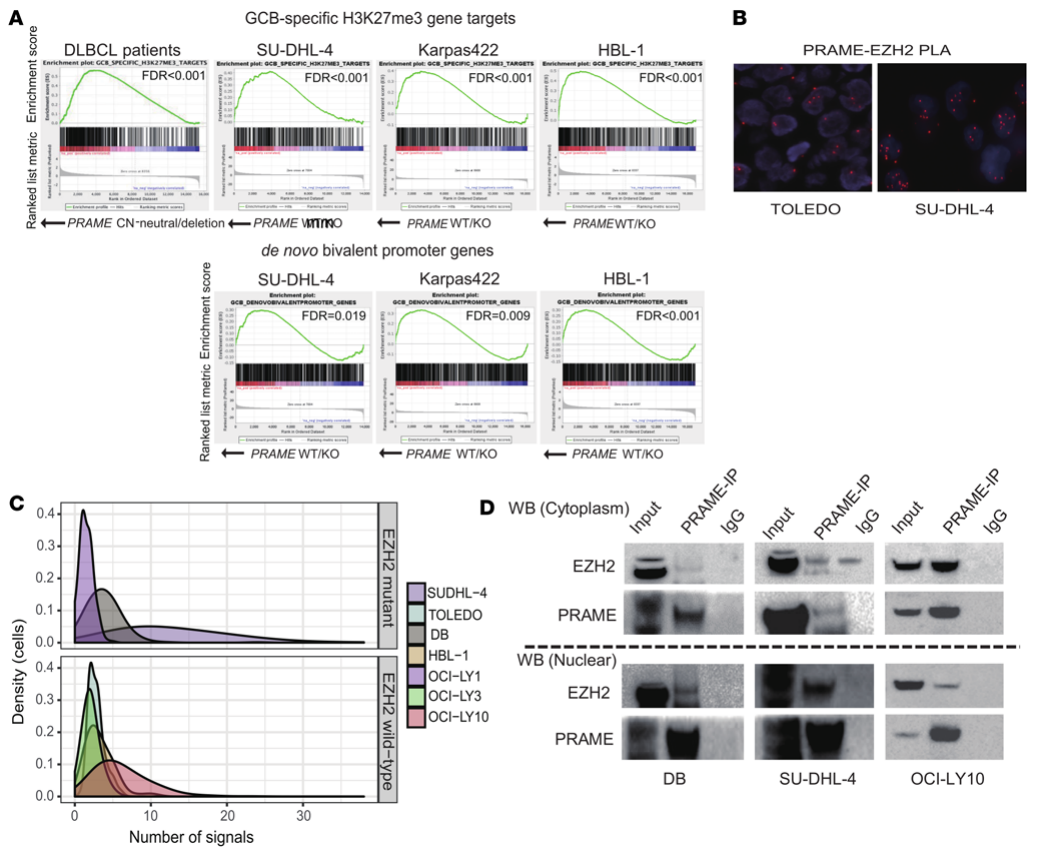
Direct interaction between PRAME and EZH2 and regulated downstream expression programs. (**A**) Preranked GSEA for enrichment of H3K27me3 target genes and de novo bivalent promoter genes for *PRAME*-CN-neutral versus *PRAME*-deleted samples in DLBCL patient samples and cell lines (SU-DHL-4, Karpas-422, and HBL-1). (**B**) Representative PLA results in SU-DHL-4 (right) and TOLEDO (left) cells. (**C**) Summary of PLA results according to *EZH2* mutation status. The density of cells (*y* axis) is shown according to number of PLA signals observed (*x* axis). (**D**) Co-IP assay in DB cells (left), SU-DHL-4 cells (middle), and OCI-LY10 (right). Cells were fractionated into cytoplasmic and nuclear components. Upper panel indicates EZH2 immunoblotting, and lower panel indicates PRAME immunoblotting.

We next sought to elucidate potential intrinsic, cell-autonomous roles of PRAME. Given that the data presented above suggest a functional interaction of PRAME and EZH2, we next focused on EZH2 activity differences between *PRAME*-CN-neutral and *PRAME*-deleted patient samples. We found that PRAME KO induced an increased repression of PRC2 target genes in the EZH2-mutated cell lines Karpas-422 and SU-DHL-4 (Figure 7A). Moreover, to address the independence of PRAME loss from *EZH2* mutation status, mRNA-Seq was performed in HBL-1 (*EZH2*-WT) cells, and we performed GSEA using the same gene sets (H3K27me3 gene targets and de novo bivalent promoter genes; ref. 28). Importantly, we found the same de-enrichment of gene targets and a shift of de novo bivalent promoter genes in HBL-1 (Figure 7A), suggesting that PRAME loss–associated effects were independent of *EZH2*-mut status. From these observations, we conclude that PRAME can act as an inhibitor of PRC2/EZH2 function and that both *EZH2* and *PRAME* can regulate each other in a regulatory loop. Furthermore, GSEA identified gene targets in the apoptosis pathway that were de-enriched in PRAME-KO cells (Supplemental Figure 13). Previous literature demonstrated a direct interaction between PRAME and PRC 2/EZH2 in HEK293 cells and repression of downstream signaling (34). Therefore, we interrogated whether direct interaction of PRAME and EZH2 also occurred in DLBCL tumor cells. We screened 7 DLBCL cell lines (*EZH2*-mut DLBCL: SU-DHL-4, DB, and OCI-LY1; *EZH2*-WT DLBCL: HBL-1, OCI-LY3, TOLEDO, and OCI-LY10) by proximity ligation assays (PLAs). SU-DHL-4 and DB (*EZH2* mut) as well as OCI-LY10 (*EZH2* WT) showed strong colocalization signals, whereas the other cell lines showed only moderate colocalization patterns compared with negative control cells (Figure 7, B and C, and Supplemental Figure 14). Co-IP of subcellular fractions (cytoplasmic and nuclear) confirmed direct interaction between PRAME and EZH2, with more pronounced interactions seen in the nucleus as compared with the cytoplasmic fraction in the *EZH2*-mut cell lines DB and SU-DHL-4 (Figure 7D). Next, to investigate whether PRAME downregulation affects EZH2/PRC2 activity, we examined the change of H3K27me3 and me2 in 2 PRAME–isogenic KO cell line systems (Karpas-422 and SU-DHL-4). *PRAME*-KO cell lines showed an increase of H3K27me3 modifications compared with native PRAME cell lines, establishing PRAME as a negative regulator of PRC2 activity and related changes in expression programs (Supplemental Figure 15).

## Discussion

In this study, we showed that PRAME was involved in dual functions (cell extrinsic and intrinsic) during lymphoma pathogenesis. We observed that PRAME downmodulation was mainly caused by *PRAME* deletion or epigenetic regulation and triggered a cold status of the TME in conjunction with antiapoptotic effects. Importantly, EZH2 inhibition can dynamically change these dual effects by restoration of PRAME and perhaps other factors. The cancer testis antigen family of proteins consists of nearly 225 genes, and excitement about cancer testis antigens has reemerged with the discovery of the effectiveness of cellular immune therapies targeting TME biology (17). In particular, our demonstration of dual functions of PRAME resulting in potentially synergistic therapeutic reversion of PRC2-mediated and immune escape mechanisms appears attractive in a subset of patients with lymphoma.

Focusing on the extrinsic effects of PRAME expression and peptide presentation, we observed downregulation of immune response and inflammatory response pathways in *PRAME*-deleted patient samples. CCL22 and CCL17 were significantly downregulated cytokines in *PRAME*-deleted tumor samples, emphasizing the role of PRAME in TME crosstalk. Specifically, these cytokines are known to contribute to increased infiltration of T follicular helper cells and/or Tregs in lymphoma and solid cancers (35–37). Although the mechanisms of differential effects on CD4^+^ and CD8^+^ T cells are still to be elucidated, these results provide evidence of a cold TME status in *PRAME*-deleted patient samples. Peng et al. demonstrated multiple EZH2-mediated effects on the TME, such as increasing levels of the Th1 type chemokines, CXCL9 and CXCL10 (38). As such, our murine model cannot exclude the possibility that EZH2i leads to direct effects in immune cells of the TME independent of PRAME status. However, this treatment model provides additional in vivo evidence that “epigenetic switches” can contribute to turning the microenvironment to a “hot” status, providing a rationale for combined epigenetic/immune based therapies that are currently under investigation (39). Interestingly, we also observed an increase of Tregs together with other T cell subsets in the TME of the mouse model, and it remains to be determined whether Tregs act as a mediator of immunosuppression subtracting from an immunologically hot status or Tregs are part of a more global inflammatory TME response. Supporting the latter possibility, our group has previously described in primary DLBCL samples that a cold TME, caused by MHC I/II downregulation, is characterized by coordinated depletion of Tregs, NK/T cells, and cytotoxic T cells in DLBCL (18).

Memory T cell reaction to PRAME antigens from patient-derived PBMCs has been reported in chronic myeloid leukemia and myeloid leukemia but not in any types of lymphoma to our knowledge, including DLBCL (23, 32, 40). Our present study demonstrated that only PBMCs from patients with PRAME-deleted tumors showed PRAME peptide reactivity. This model indicates that PRAME expression increases during malignant transformation, creating T cell memory in a subset of cases. As a second step in this setting, tumors escape T cell detection by deletion of the *PRAME* gene locus with *PRAME*-deleted tumor cells, thus avoiding cytotoxic T cell attack. In other words, PRAME-deleted tumors might be “preeducated” for cytotoxic T cell activity specific to PRAME antigens. These results are in agreement with Rezvani et al., who demonstrated CD8^+^ T cell responses in patients with leukemia while healthy donors showed only a weak response to 1 PRAME peptide (PRA 300; ref. 32).

Addressing cell-intrinsic regulatory pathways, we focused on *EZH2* hotspot mutations that were strongly associated with PRAME downmodulation. EZH2 is a core component of PRC2 that methylates lysine 27 of histone 3 to generate the repressive H3K27me3 histone mark (41, 42). Given that a direct interaction of PRAME with PRC2 has been previously shown in HEK293 cells (34), we tested PRAME-EZH2 interaction across a spectrum of DLBCL-derived cell lines and confirmed direct protein interaction irrespective of *EZH2* mutation status, which is consistent with a function of PRAME as a negative regulator of PRC2. Focusing on specific target genes that are known to be regulated by EZH2 in the context of PRAME expression, we found that apoptotic pathway genes were significantly downregulated in *PRAME*-KO cell lines (Karpas-422 and SU-DHL-4) compared with *PRAME* WT (Supplemental Figure 13 and Supplemental Table 8). EZH2 inhibition might disrupt the autoregulatory loop involving PRAME and induce cell death via activation of apoptotic signaling. Furthermore, our GSEA results in a *PRAME*-KO cell line model with *EZH2* WT (HBL-1) indicated that PRAME can act as a negative regulator of PRC2 independent of EZH2 mutation status.

Our study outlines potentially new therapeutic choices for patients with intractable DLBCL. Recently, generation of cytotoxic T cells targeting PRAME antigens for the treatment of acute myeloid leukemia, chronic myeloid leukemia, and medulloblastoma has been reported (23, 24, 32). Use of PRAME-specific cytotoxic T cells might be a strategy for a sizable subgroup of patients with DLBCL exploiting the concept of EZH2 inhibition–mediated enhancement of PRAME expression and TAA presentation. This might be combined with other regulators of PRAME expression (22). In particular, our data suggest that PRAME restoration might be most effective in patients with preexisting anti-PRAME memory T cell responses.

In summary, we have revealed dual functions of PRAME in DLBCL, providing therapeutic rationales for treatment of high-risk patient populations in DLBCL, other lymphomas, and cancers in general that rely on immune escape phenotypes.

## Methods

### Patient cohort description.

Initially, the BCCA Lymphoid Cancer database was searched to identify all patients with DLBCL diagnosed between 1985 and 2011. From 4063 DLBCL cases, 347 patients with de novo DLBCL were included in the final cohort for analysis if they met the following criteria: patients had to be 16 years of age or older; had to be treated uniformly with R-CHOP with curative intent at British Columbia Cancer; had complete clinical, laboratory, and outcome data available; and had a fresh-frozen diagnostic biopsy. The diagnosis was made according to the 2008 WHO classification, as determined by standardized review by expert hematopathologists (43). Patients were excluded if they had any of the following: primary mediastinal large B cell lymphoma; primary or secondary CNS involvement at diagnosis; a previous diagnosis of an indolent lymphoproliferative disorder; positive HIV serology; or a secondary malignancy or major medical comorbidity that precluded treatment with curative intent. As an independent validation cohort, we also selected 52 de novo patients with DLBCL diagnosed between 2013 and 2016 for which *PRAME* CN status was available from a published study (31).

### SNP6.0-based CN analysis.

We performed SNP6.0 arrays for CN analyses using fresh-frozen samples from 341 DLBCL cases as previously described (19). Library construction and data processing were previously described (44, 45). Briefly, we used the OncoSNP pipeline to provide CN segments and gene-centric CN states. We also ran GISTIC (v2.0.12) on the OncoSNP segmented data to identify minimally commonly deleted and amplified regions.

### Survival analysis.

The Kaplan-Meier method was used to estimate the TTP (progression/relapse or death from lymphoma or acute treatment toxicity), PFS (progression/relapse or death from any cause), DSS (death from lymphoma or acute treatment toxicity), and overall survival (death from any cause), with log-rank tests performed to compare survival curves. In this study, we mainly used TTP to reflect the influence of genetic features on tumor progression by removing the death event not caused by lymphoma. Univariate and multivariate Cox proportional hazard regression models were used to evaluate proposed prognostic factors.

### HLA subtyping from RNA-Seq.

HLA typing was performed for cell lines using OptiType (v1.3.1; ref. 46) and seq2HLA (v2.2; ref. 47) using whole-transcriptome RNA-Seq from DLBCL cell lines as input. Results from both tools were concordant. HLA typing was performed with RNA-Seq data from patients with DLBCL using OptiType (v1.2) with default parameters.

### IHC on tissue microarray and flow cytometry.

For IHC staining, 4 μm slides of tissue microarrays from 341 DLBCL cases and antibodies listed in Supplemental Table 9 were used. Staining was performed on a Benchmark XT platform (Ventana). The protein expression of PRAME was recorded semiquantitatively; score 0: negative, score 1: weak positive, score 2: strong positive. Immunohistochemically stained slides for the T cell markers CD4, CD8, FOXP3, PD-1, GATA3, and T-bet as well as the macrophage markers CD68 and CD163 were scanned with an Aperio ScanScope XT at 20× original magnification. Image analysis was performed using the ImageScope viewer (v12.1.0; Aperio Technologies). The positive pixel count algorithm with an optimized color saturation threshold was then applied to tumor-containing areas, and any staining was considered positive. The number of positive pixels was divided by the total pixel count and multiplied by 100 to obtain the percentage of positive pixels. Representative images were taken using a Nikon Eclipse 80i microscope equipped with a Nikon DS-Ri1 camera and NIS Elements imaging software, D3.10.

### RNA-Seq, gene expression, GSEA, and pathway analysis.

RNA-Seq data were generated from fresh-frozen tissue of 322 patients with DLBCL on the Illumina HiSeq 2500 platform as previously described (18). RNA-Seq of the patient samples was performed using bulk mRNA from frozen tissues. The sequencing data were aligned to GRCh37-hg19 with the STAR aligner (v2.5.2a), which also produced per-gene count data with HTSeq-Count. The STAR metrics were used to assess the total number of uniquely mapped reads in each of the 322 cases. A threshold of the mean value minus 2 standard deviations was calculated (24,137,346 reads), and 8 cases with fewer uniquely mapped reads than the threshold were removed from analysis. Of the remaining cases, PRAME deletion status was successfully assessed in 311 cases.

Analysis of the data was performed in R (v3.4.4). Raw counts for the 311 cases were read using the DESeq2 package (v1.18.1), and only genes with transcripts per million reads 1 or more in at least 5% of the cases were retained (15,486 genes). Differential expression analysis was performed between cases harboring a PRAME deletion (*n* = 43) and those with WT PRAME (*n* = 268), as well as EZH2 mutation (*n* = 46) or WT (*n* = 301). Significantly differentially expressed genes from this comparison were selected (adjusted *P* < 0.05, absolute log_2_ fold change ≥ 1), and enriched pathways in these genes were identified using the Database for Annotation, Visualization and Integrated Discovery functional annotation tool (v6.8; ref. 48) with annotations from Gene Ontology, BioCarta, and Kyoto Encyclopedia of Genes and Genomes (*Homo sapiens* annotation only). In addition, GSEA Preranked (v4.0.2) was used to assess enrichment of specific pathways using the log_2_ fold change between PRAME-deleted and WT cases for gene ordering.

### RNA-Seq cell line analysis.

PRAME heterozygous and homozygous deletions were introduced into 3 DLBCL cell lines, SU-DHL-4, Karpas-422, and HBL-1. For the SU-DHL-4 and Karpas-422 cell lines, RNA-Seq was performed for 3 conditions: PRAME WT clones (*n* = 3, 2 technical replicates each), PRAME heterozygous-deleted clones (*n* = 2, 2 technical replicates each), and PRAME homozygous-deleted clones (*n* = 1, 2 technical replicates each). For the HBL-1 cell line, RNA-Seq was performed for 2 conditions: PRAME WT clones (*n* = 3, 2 technical replicates, each), and PRAME homozygous-deleted clones (*n* = 3, 2 technical replicates each). RNA-Seq was performed on an Illumina HiSeq 2500 (paired-end 75 bp reads). Reads were aligned to the GRCh37 reference using the STAR aligner (v2.5.3a_modified), which also generated per-gene count data with HTSeq-Count. Raw counts were read into R (v4.1.0), and only genes with a total count of 10 or more across all samples were retained (22,723 genes). Differential expression was performed between all PRAME WT clones (SU-DHL-4, Karpas-422, and HBL-1) and all PRAME-deleted clones (heterozygous and homozygous) with DESeq2 (v1.18.1). GSEA Preranked (v4.1.0) was used to examine specific pathways between the 2 types with the *P* value as the ranking feature. RNA-Seq data were deposited in NCBI’s Gene Expression Omnibus (GSE190403).

### Cell lines.

Human DLBCL cell lines Karpas-422, SU-DHL-4, WSU-DLCL2, SU-DHL-10, SU-DHL-6, DB, DOHH-2, HT, and SU-DHL-8 were purchased from DSMZ. The TOLEDO cell line was purchased from ATCC. OCI-LY1 and OCI-LY3 were provided by the Louis M. Staudt lab (Center for Cancer Research, National Cancer Institute, NIH, Bethesda, Maryland, USA); HBL-1 and OCI-LY10 were provided by the Michael Gold lab (Department of Microbiology and Immunology, University of British Columbia, Vancouver, British Columbia, Canada). Karpas-422, SU-DHL-4, SU-DHL-10, SU-DHL-8, SU-DHL-6, and DB were cultured in RPMI 1640 (Thermo Fisher Scientific) supplemented with 20% FBS (Thermo Fisher Scientific). WSU-DLCL2, DOHH-2, OCI-LY1, HT, HBL-1, and TOLEDO were cultured in RPMI 1640 supplemented with 10% FBS. OCI-LY1 and OCI-LY10 were cultured in IMDM supplemented with 10% and 20% FBS, respectively. All cell lines were confirmed to be negative for mycoplasma using Venor GeM Mycoplasma Detection Kit, PCR-based (MilliporeSigma, MP0025). All cell lines were authenticated by STR profiling (The Centre for Applied Genomics, The Hospital for Sick Children, Toronto, Canada, Supplemental Table 10). Mutations in *EZH2* in the cell lines were evaluated using the COSMIC (https://cancer.sanger.ac.uk/cosmic) databases and in-house whole-genome or whole-exome sequencing data.

### Gene editing.

Human PRAME-KO cells (PRAME heterozygous or homozygous KO) were generated using the CRISPR/Cas9 (CRISPR-associated) system. Target sequences were found in exon 4 of human PRAME, transcript variant 202 (NCBI: NM_206956.3), using the CRISPOR design tool (CRISPOR, http://crispor.tefor.net/) as described (49). We synthesized 3 kinds of gRNA for CRISPR gene editing using Alt-R methods (Integrated DNA Technologies): 5′ CTGGCTGTGTCTCCCGTCAA 3′; 5′ CCAGCTCCACAAGTCTCCGT 3′; 5′ CAGCAACTCCAGGGCGGCAA 3′.

Ribonucleoprotein complex was introduced into the Karpas-422, SU-DHL-4, and HBL-1 cell lines by nucleofection using the Lonza Nucleofector II electroporation system. Cells were plated for 0.5 cells per well in 96-well plate after 24 hours of nucleofection using Methocult H4434 media (STEMCELL Technologies). To identify clones carrying mutations within PRAME, genomic DNA was prepared from expanded individual clones with Quick DNA mini-prep kit (Zymo Research), and the CRISPR target site was amplified for Sanger sequencing using primers (5′ CAGGTGCATGTTCCTTCAGA 3′; 5′ AGCCCTCAGGCTCCTTAGTT 3′). Colonies carrying monoallelic and/or biallelic changes resulting in frameshifting indels were further verified by cloning amplified CRISPR target sites into TOPO TA vectors (Invitrogen) followed by Sanger sequencing of each allele.

### EZH2 knockdown assay.

WSU-DLCL2 cells were transduced by lenti-GFP-shEZH2 (OriGene TL304713), and GFP-positive cells were enriched using a FACSAria III cell sorter (BD Biosciences). Western blotting was performed using EZH2 (H.547.3, Thermo Fisher Scientific), PRAME (EPR20330, Abcam), H3K27me3 (Lys27, C36B11, Cell Signaling Technology), and GAPDH (MAB374, MilliporeSigma) antibodies.

### In vitro coculture assay.

PBMCs from healthy human donors were obtained from Miltenyi Biotec, and naive CD4^+^ cells were negatively selected using the naive CD4^+^ T cell isolation kit II by MACS according to the manufacturer’s instructions. CD4^+^ cells were activated using anti-CD3 (clone OKT3, 5 μg/mL) and anti-CD28 (clone CD28.2, 10 μg/mL) mAb–precoated 96-well plates. Next, 2 × 10^5^ T cells and 4 × 10^3^ DLBCL cells (SU-DHL-4 or Karpas-422) were cocultured at a 50:1 ratio in AIM-V medium (Invitrogen) supplemented with HLA-DR/DP/DQ mAb (clone WR18, 10 μg/mL) for MHC II blockade for 3 days. T cell proliferation was assessed using CellTrace violet dye (Invitrogen). Prior to coculturing, CD4^+^ cells (1 × 10^7^ cells/mL) were stained with 5 μM CellTrace violet dye and incubated at 37°C for 15 minutes with gentle vortexing every 5 minutes. Flow cytometry was performed on the FACSSymphony flow cytometer (BD Biosciences) using the following Abs/stains: LIVE/DEAD Yellow Fixable dye 1:1000 (Invitrogen), CD4 BV786 clone SK3 1:200 (BD Biosciences), CD8 APC-H7 clone SK1 1:200 (BD Biosciences), and CD69 FITC clone FN50 1:50 (BioLegend).

### In vitro T cell priming and IFN-γ ELISPOT assay.

Monocyte-derived DCs were pulsed with individual PRAME peptides (PRA100: VLDGLDVLL, PRA142: SLYSFPEPEA, PRA300: ALYVDSLFFL, PRA425: SLLQHLIGL) and cultured with autologous PBMCs to active peptide-specific CD8^+^ T cells as described (50). PBMCs derived from 4 *PRAME*-WT, 4 *PRAME*-deleted, and 7 healthy donors were used for this assay. After a second round of peptide stimulation, T cells were screened for reactivity by IFN-γ ELISPOT. ELISPOT plates were read using an AID EliSpot Reader and quantified using AID EliSpot 7.0S software (AutoImmun Diagnostika). At least 3 replicates of T cell/well were counted and PRAME-peptide/nonpeptide number ratio was calculated.

### EZH2i treatment assay, Western blotting, and Pr20 binding assay.

DLBCL cell lines were grown in 24-well plates, and cell viability was determined using the trypan blue automatic method (Countess II FL automated cell counter, Thermo Fisher Scientific). DLBCL cell lines were exposed at 6 concentrations (DMSO: 0 μM, 0.1 μM, 0.5 μM, 1 μM, 2 μM, 5 μM) of tazemetostat (EPZ-6438) for 4 days and analyzed for cell viability as before. After 4 days of treatment, cells were harvested and cell lysates were prepared using RIPA buffer (Thermo Fisher Scientific), and total proteins were quantified using a BCA protein assay kit (Thermo Fisher Scientific). NuPAGE Novex Bis-Tris gradient gel (4%–12%) (Invitrogen) was used for protein loading (25 μg each sample) and transferred to a 0.45 μm nitrocellulose membrane (Bio-Rad) by semidry transfer. Blots were stained with anti-PRAME (EPR20330, Abcam), anti-H3K27me3 (Lys27, C36B11, Cell Signaling Technology), and anti-EZH2 (H.547.3, Thermo Fisher Scientific) following the manufacturer’s instructions. GAPDH (MAB374, MilliporeSigma) was included as an internal loading control. HRP-conjugated anti-rabbit or anti-mouse IgG (Promega) was used to visualize labeled proteins. ECL system (GE Life Sciences) on a Chemidoc digital imager (Bio-Rad) was used for protein detection. For the Pr20 mAb binding assay, DLBCL cell lines were incubated with Pr20 mAb or its isotype control human IgG1 mAb (3 μg/mL) for 30 minutes on ice and washed in PBS. Analysis was done by flow cytometry on a Beckman Dickinson Fortessa and analyzed with FlowJo 9.8.1 and FlowJo 10 software.

### ChIP assay and qPCR.

ChIP was conducted using the SimpleChIP Chromatin IP kit (Cell Signaling Technology) in DLBCL cells (DOHH-2, HT, SU-DHL-8, HBL-1, DB, Karpas-422, SU-DHL-10, WSU-DLCL2, SU-DHL-6, and SU-DHL-4) according to the manufacturer’s protocol. Primers for the *PRAME* promoter region were designed according to the ENCODE transcription binding site as previously described (51). Real-time PCR was executed with purified DNA in the Power SYBR Green PCR system (Invitrogen) according to the manufacturer’s protocol.

### PLA.

PLA was performed as previously described (52, 53). Briefly, DLBCL cells were fixed with 10% formaldehyde and embedded into paraffin tissue blocks. Samples were incubated with primary antibodies (rabbit anti-PRAME antibody: Abcam EPR20330 and mouse anti-EZH2 antibody: Thermo Fisher Scientific 144CT2.1.1.5) for 1 hour at 37°C. PLA probes (Duolink In Situ PLA Probe Anti-Mouse MINUS and Anti-Rabbit PLUS, MilliporeSigma) were incubated for 1 hour at 37°C. Then, ligation (30 minutes at 37°C) and amplification steps (100 minutes at 37°C, Duolink In Situ Detection Reagents Orange) were performed. The fluorescent signals were analyzed by Metafer4 (v3.11.8) using Zeiss Imager.Z2 microscopy.

### Co-IP assay.

DLBCL cells (SU-DHL-4, DB, and OCI-LY10) were lysed using lysis buffer (10 mM Tris-HCl pH8, 10 mM NaCl, 3 mM MgCl_2_, 0.5% NP-40) and nuclei were collected on ice. CHAPS buffer (FIVEphoton Biochemicals) was used for nuclear extraction, and the lysate was incubated with 2 μg of PRAME (Abcam, EPR20330) antibodies overnight at 4°C on a rocker. Protein A/G beads (Thermo Fisher Scientific) were added (20 μL), and samples were rotated for 1 hour at 4°C. Beads were collected by centrifugation and extensively washed with CHAPS lysis buffer. Proteins were eluted into SDS gel loading buffer by heating at 65°C. Proteins were separated using NuPAGE 4% to 12% gels (Thermo Fisher Scientific), transferred to PVDF membranes, and stained as described in *EZH2i treatment assay, Western blotting, and Pr20 binding assay*.

### Murine lymphoma model treated with EZH2i.

*Ezh2*(Y641F)^fl/+^ mice were generated as described in Béguelin et al. (33). IμBcl6 mice were obtained from Ricardo Dalla-Favera, Columbia University, New York, New York, USA (54). We crossed IμBcl6 with *Ezh2*(Y641F)^fl/+^ Cγ1-Cre mice to engineer BCL6 constitutive expression and mutant EZH2 activity in GCB cells. The bone marrow of these mice was next transplanted into lethally irradiated recipient C57BL/6J mice from The Jackson Laboratory (1 × 10^6^ cells per recipient). Animals were immunized with sheep red blood cells every 3 weeks to ensure continuous formation of GCs and were observed for spleen growth. Three months after the bone marrow transplant, mice were treated with vehicle (0.5% sodium carboxymethyl cellulose + 0.1% Tween 80) or EZH2i EPZ011989 at 250 mg/kg (bid) by oral gavage administration for 28 consecutive days. Three mice per group were euthanized at 3 time points: right after treatment and 3 months and 4 months after treatment withdrawal. Spleen, submandibular lymph nodes, lung, liver, and kidney were collected and processed for formalin fixing and paraffin embedding.

### Statistics.

Statistical analyses were performed using 1-way ANOVA with Bonferroni’s correction for multiple comparisons or unpaired 2-tailed *t* test. A *P* value less than 0.05 was considered statistically significant in all experiments.

### Study approval.

Human studies were reviewed and approved by the University of British Columbia British Columbia Cancer Research Ethics Board (H14-02304: Biomarkers in lymphoid cancer and H17-01829: PRAME deletions in DLBCL), in accordance with the Declaration of Helsinki. We obtained written informed consent from the patients, or informed consent was waived for the samples used in this retrospective study. All animal experiments were approved by the Research Animal Resource Center of the Weill Cornell Medical College (IACUC 2011-031: Molecular and epigenetic mechanisms of lymphoma genesis).

## Author contributions

KT designed and performed experiments, analyzed the results, and wrote the manuscript. LCC, DE, AT, SH, and EV performed experiments, analyzed the results, and wrote the manuscript. SBN, TA, ET, MYL, SSH, MB, BW, AT, AWZ, AB, DK, GD, MK, SR, and SS performed experiments and analyzed the results. TD, SSM, CMB, and DAS performed the Pr20 binding assay. JSN, GDA, and BHN supervised and analyzed ELISPOT assays. AMM and WB generated the *Ezh2*(Y641F)^fl/+^ Cγ1-Cre mice model treated with EZH2i and contributed to writing the manuscript. SPS, PAH, RDG, JMC, and DWS provided guidance and novel tools of the project and contributed to writing the manuscript. CS supervised and designed the study and wrote the manuscript.

## Supplementary Material

Supplemental data

Supplemental tables 1-10

## Version 1 04/05/2022

In-Press Preview

## Version 2 05/16/2022

Electronic publication
