# Supplementary material for: Tumor-associated antigen PRAME exhibits dualistic functions that are targetable in diffuse large B cell lymphoma
Source: J Clin Invest. 2022 May 16;132(10):e145343. doi: 10.1172/JCI145343 (PMC9106353; doi:10.1172/JCI145343)
Supplement: Supplemental data [file jci-132-145343-s090.pdf]

Supplementary Materials

The tumor associated antigen PRAME exhibits dualistic functions that are targetable in diffuse large B cell lymphoma

Katsuyoshi Takata[1,2], Lauren C. Chong[1], Daisuke Ennishi[1], Tomohiro Aoki[1], Michael Yu Li[1], Avinash Thakur[3,4], Shannon Healy[1], Elena Viganò[1], Tao Dao[5], Daniel Kwon[6], Gerben Duns[1], Julie S. Nielsen[7], Susana Ben-Neriah[1], Ethan Tse[1], Stacy Hung[1], Merrill Boyle[1], Sung Soo Mun[5], Christopher Bourne[5], Bruce Woolcock[1], Adèle Telenius[1], Makoto Kishida[1], Shinya Rai[1], Allen Zhang[8], Ali Bashashati[8], Saeed Saberi[8], Gianluca D' Antonio[7], Brad H. Nelson[7], Sohrab P. Shah[8,9], Pamela A. Hoodless[3,4], Ari M. Melnick[10], Randy D. Gascoyne[1], Joseph M. Connors[1], David A. Scheinberg[5], Wendy Béguelin[10], David W. Scott[1], Christian Steidl[1,11]*

Correspondence to: CSteidl@bccancer.bc.ca

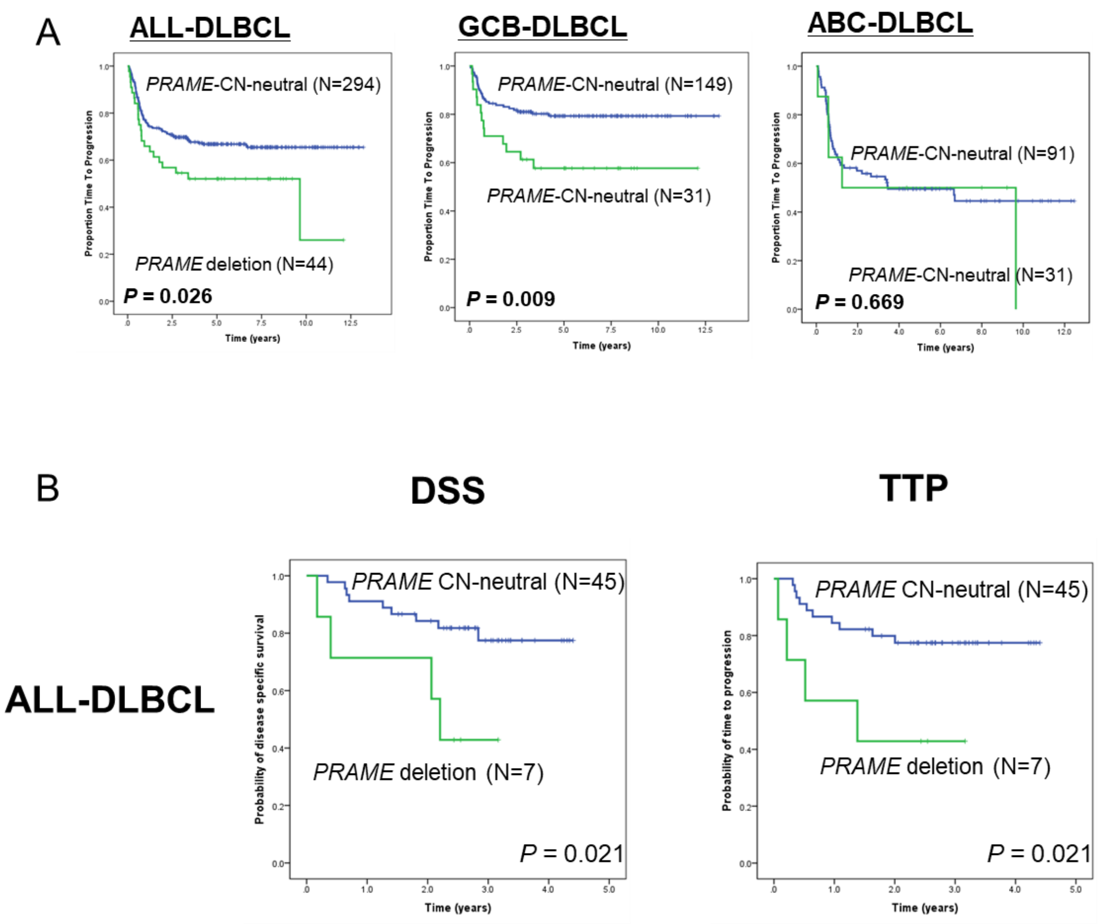

Figure.S1. Kaplan-Meier analysis of discovery cohort and validation cohort. (A) Time to Progression (TTP) survival in all- (left), GCB- (middle), and ABC-DLBCL (right). (B)
Disease specific survival (DSS) and TTP in all-DLBCL in an independent DLBCL cohort (n=52).

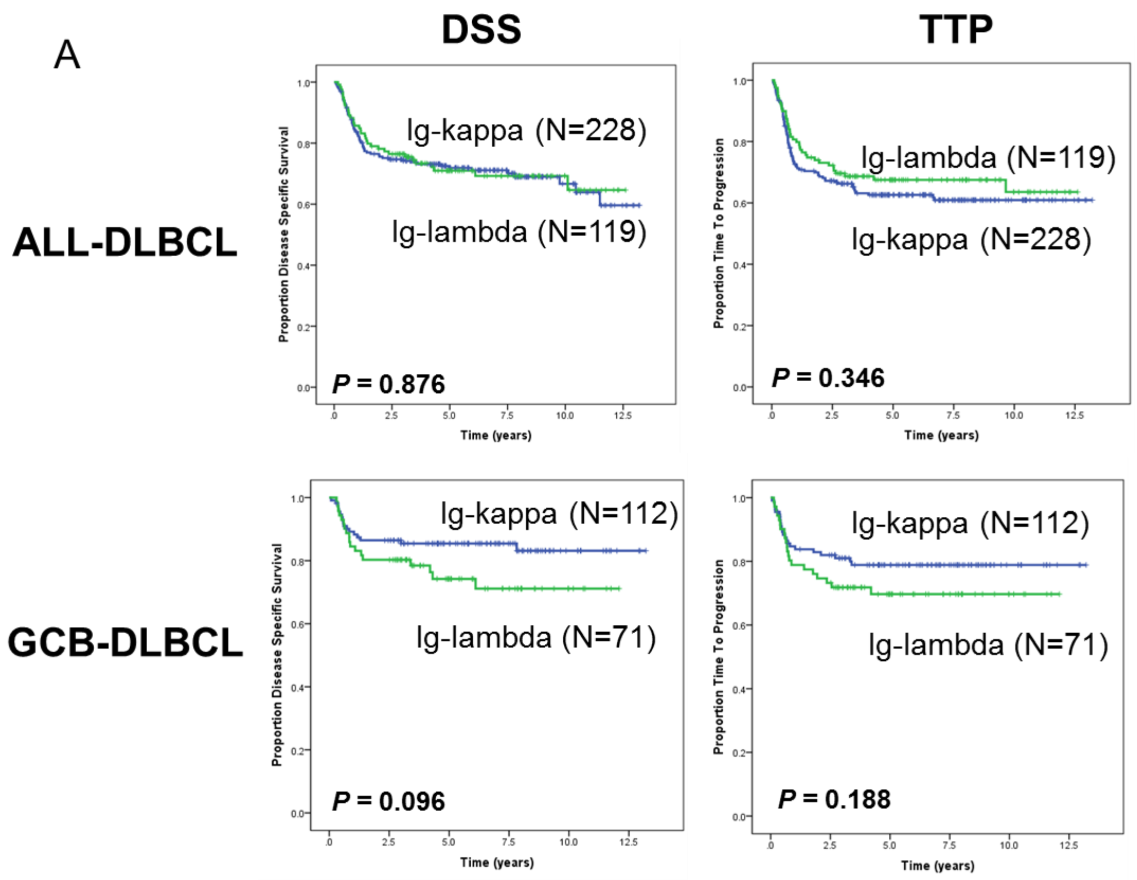
A
DSS
TTP
ALL-DLBCL
GCB-DLBCL
Ig-kappa (N=228)
Ig-lambda (N=119)
P = 0.876
Ig-lambda (N=119)
Ig-kappa (N=228)
P = 0.346
Ig-kappa (N=112)
Ig-lambda (N=71)
P = 0.096
Ig-kappa (N=112)
Ig-lambda (N=71)
P = 0.188
Proportion Disease Specific Survival
Proportion Time To Progression
Time (years)

B

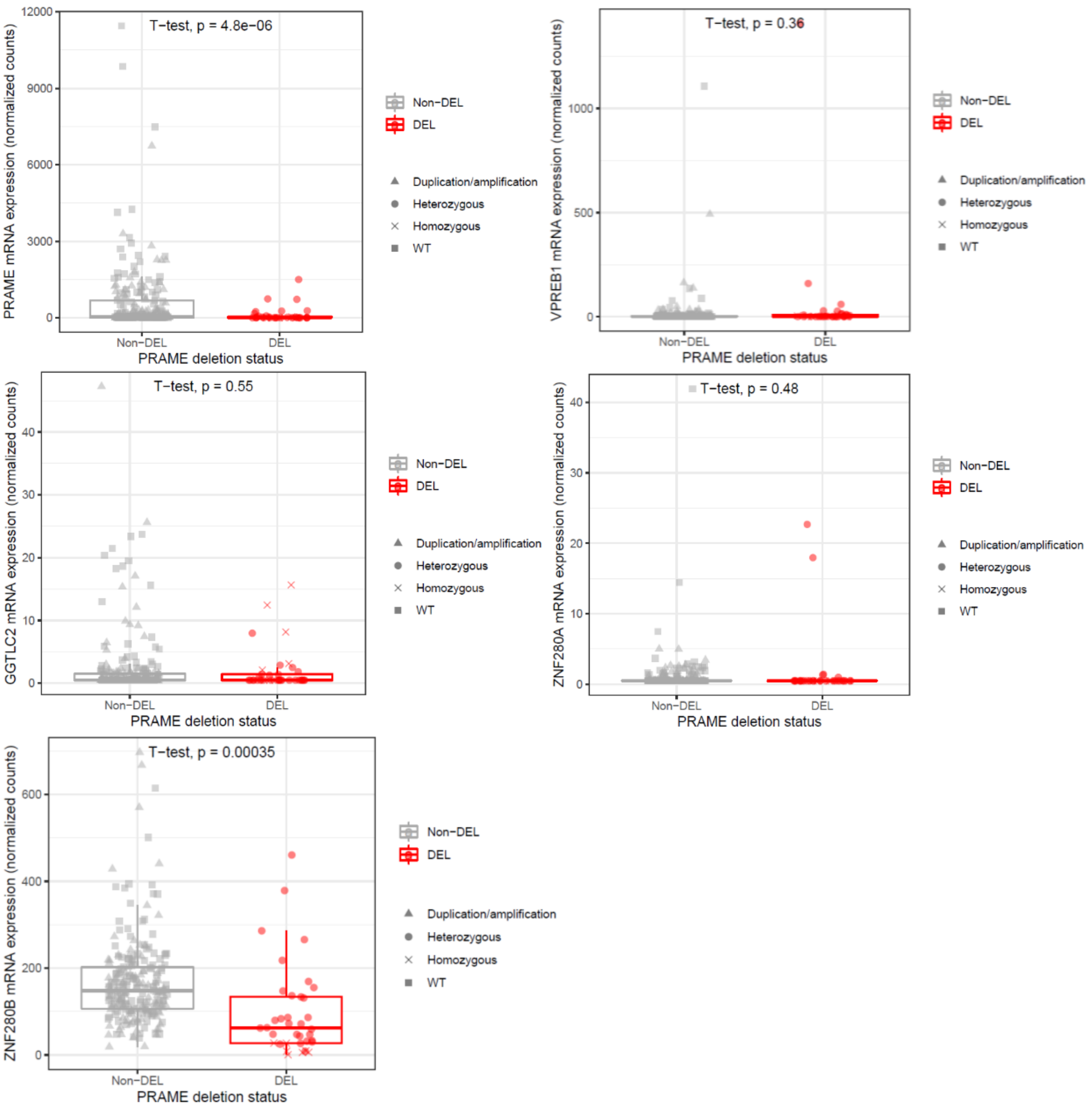
T−test, p = 4.8e−06
PRAME mRNA expression (normalized counts)
12000
9000
6000
3000
0
Non−DEL
DEL
PRAME deletion status
Non−DEL
DEL
Duplication/amplification
Heterozygous
Homozygous
WT
T−test, p = 0.36
VPREB1 mRNA expression (normalized counts)
1000
500
0
Non−DEL
DEL
PRAME deletion status
Non−DEL
DEL
Duplication/amplification
Heterozygous
Homozygous
WT
T−test, p = 0.55
GGTLC2 mRNA expression (normalized counts)
40
30
20
10
0
Non−DEL
DEL
PRAME deletion status
Non−DEL
DEL
Duplication/amplification
Heterozygous
Homozygous
WT
T−test, p = 0.48
ZNF280A mRNA expression (normalized counts)
40
30
20
10
0
Non−DEL
DEL
PRAME deletion status
Non−DEL
DEL
Duplication/amplification
Heterozygous
Homozygous
WT
T−test, p = 0.00035
ZNF280B mRNA expression (normalized counts)
600
400
200
0
Non−DEL
DEL
PRAME deletion status
Non−DEL
DEL
Duplication/amplification
Heterozygous
Homozygous
WT

C

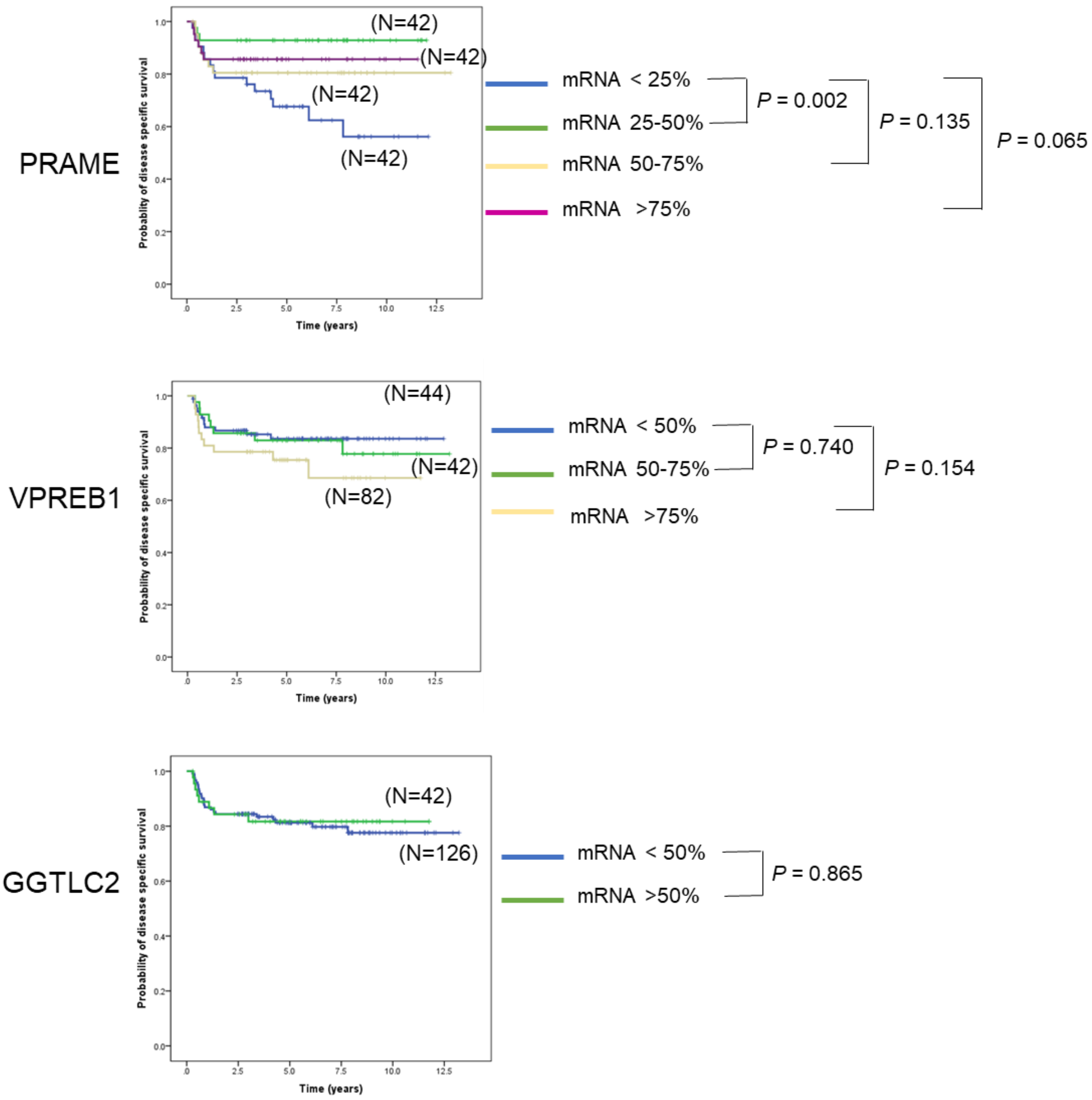
PRAME
Probability of disease specific survival
Time (years)
(N=42)
(N=42)
(N=42)
(N=42)
mRNA < 25%
mRNA 25-50%
mRNA 50-75%
mRNA >75%
P = 0.002
P = 0.135
P = 0.065
VPREB1
(N=44)
(N=42)
(N=82)
mRNA < 50%
mRNA 50-75%
mRNA >75%
P = 0.740
P = 0.154
GGTLC2
(N=42)
(N=126)
mRNA < 50%
mRNA >50%
P = 0.865

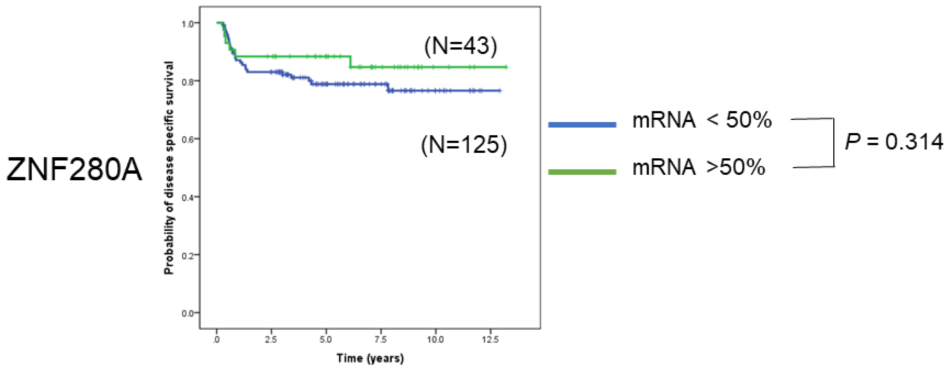

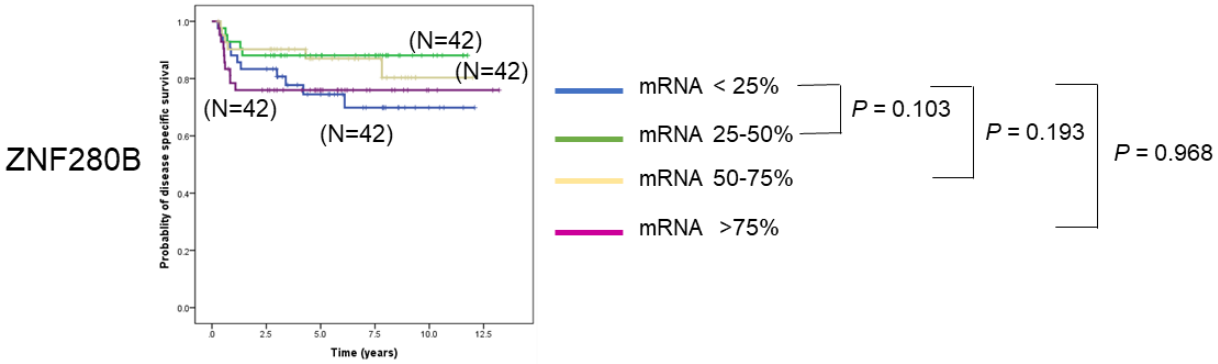

Figure. S2. Correlation analysis for the Ig-lambda and 5 genes involved in 22q11.22 deletion area.
(A) Kaplan-Meier curves represent DSS and TTP survival according to Ig-rearrangement status in all- (upper) and GCB-DLBCL (lower). (B) Cis-correlation analysis of mRNA for 5 genes *(PRAME, GGTCL2, VPREB1, ZNF280A, ZNF280B*) with *PRAME* deletion status. (C) Outcome correlation analysis using unbiased quartile mRNA cut-offs (25%, 50%, and 75%). Less than 25% of mRNA were included into <50% in VPREB1, GGTLC2, and ZNF280A because samples in the <25% and <50% quartiles showed same expression value.

A

B

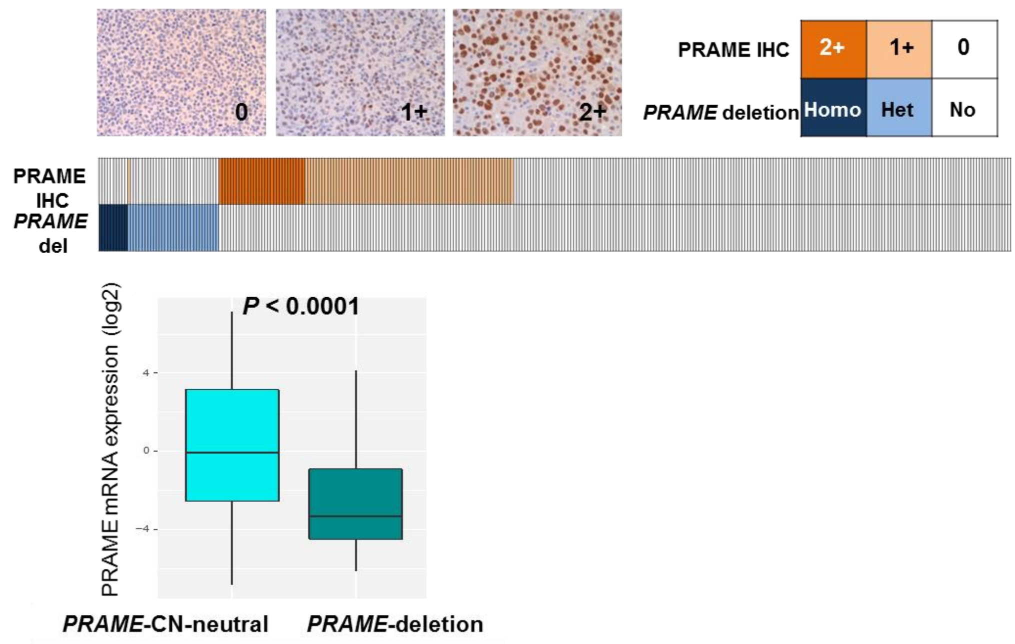

Figure.S3. Correlation with Ig-kappa, -lambda, immunohistochemistry, and mRNA. (A) Representative immunohistochemistry of PRAME and correlation with *PRAME* genetic status. (B) Correlation between *PRAME* deletion status and PRAME mRNA expression.

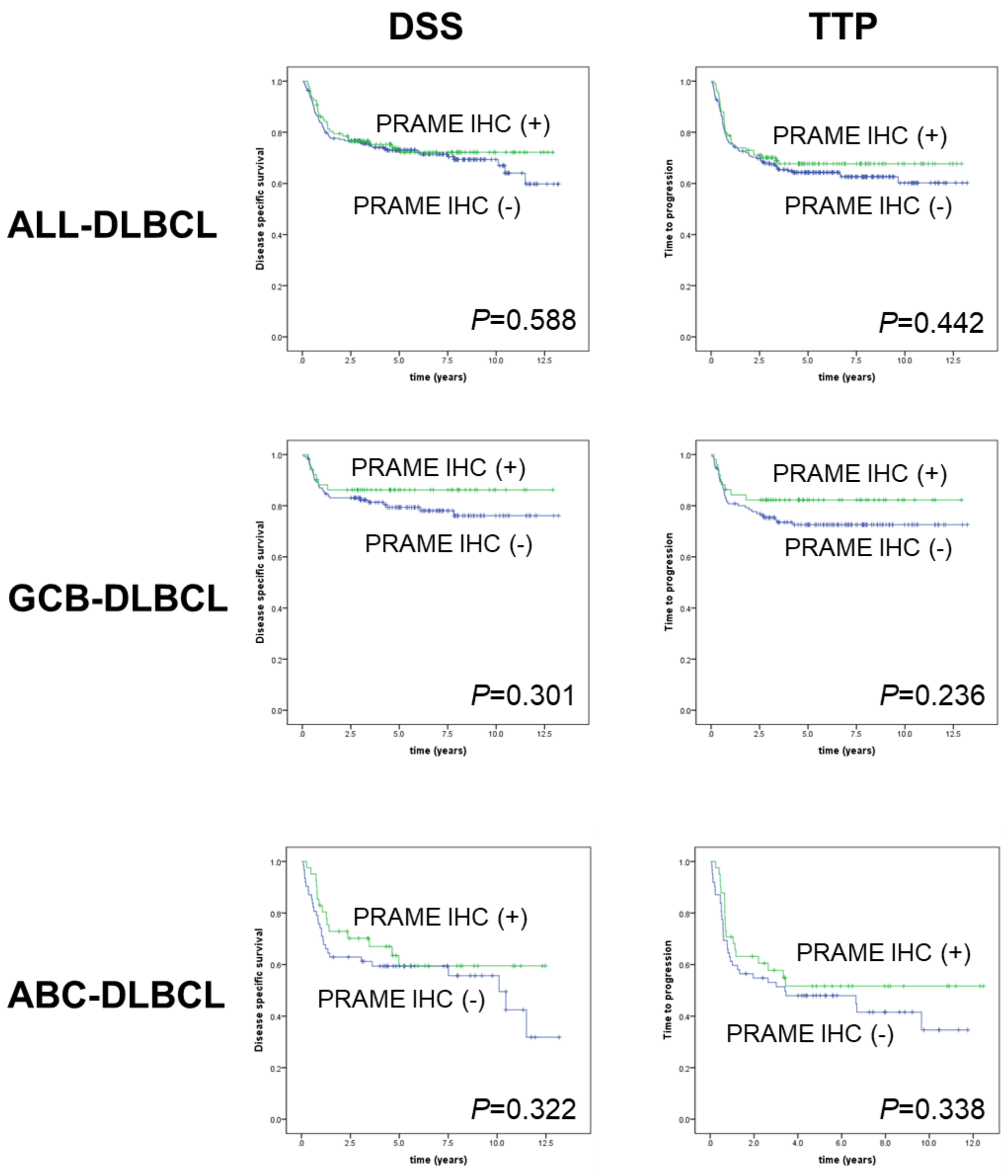

Figure.S4. Outcome correlation with PRAME IHC status.
Kaplan-Meier curves represent DSS and TTP survival according to PRAME IHC status in all- (upper), GCB- (middle), and ABC-DLBCL (lower).

A

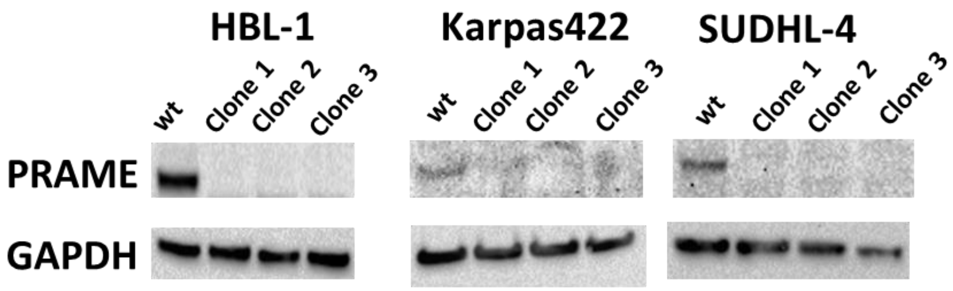

B

Cell line and clone	Sequence	Description
HBL-1 clone 1	TGTGgacaagcccacggagacttgtgGAGC	22bp frameshift
	CCCctgctgttgctagcac agtgactttggccctaagttggtccct cagaagggtgaggaaaagatagagttgcttatgcttgggctgaaag ggatgccttgttccctgtatgtttcctcagggttccattcagagccgatacat cagcatgagtgtgtggacaagcccacggagacttg tggagctggcagggcagagcCTGCT	177bp frameshift
HBL-1 clone 2	AAGtctccgtggGCTTGT	9bp frameshift
	AGCTcacaagtctccgtgggcttgtcCACACA	22bp frameshift
HBL-1 clone 3	GTCTCcgtgggcTTGTCC	7bp frameshift
	GCAGGctctgccctgccagctccacaagtctccgt gggcttgtccacacactcatgctgatgtatcggctctgaatggaaccctg aggaaacatacagggaacaaggcatccctttcagcccaagcataagcaac tctatcttttcctcacccttctgagggaccaacttagggccaaagtcact tgctagcaacagcaggggagttctcAGTTTA	238bp frameshift
Karpas-422 clone 1	CCAgctccacaagtctccgtggGCTTGTCC	19bp frameshift
	CCAGCTCCAC AAGTctccGTGGGCT	4bp frameshift
Karpas-422 clone 2	CAAgtctccGTGGGCTTGTCC	6bp frame shift
	CAAgtctccGTGGGCTTGTCC	6bp frame shift
Karpas422 clone 3	CCAgctccacaagtctccgtggGCTTGTCC	19bp frameshift
	CCAgctccacaagtctccgtggGCTTGTCC	19bp frameshift
SU-DHL-4 clone 1	AAGTctccgtgggcttgtccacacactcatgctgatgtatcggctctg aatggaaccctgaggaaacatacagggaacaaggcatcccttt cagcccaagcataagcaaCTCT	113bp frameshift
	TGCcagctccacaagtctccgtgggcttgtcCACA	28bp frameshift
SU-DHL-4 clone 2	TGCcagctccacaagtctccgtgggcttgtcCACA	28bp frameshift
	TGCcagctccacaagtctccgtgggcttgtcCACA	28bp frameshift
SU-DHL-4 clone 3	AGAGggaggcaggtgaagGGCC	14bp frameshift
	AACTTAgGGCCAA	1bp frameshift

Figure S5. Western Blotting and Sanger sequencing results for PRAME isogenic knockout clones in DLBCL cell lines. (A) Western Blotting of PRAME and GAPDH. Protein lysate (for anti-PRAME: 20μg, for anti-GAPDH: 5μg) was loaded in the separate gels and transferred to separate membranes.
(B) Summary of Sanger sequencing for PRAME exon 4 region.

A

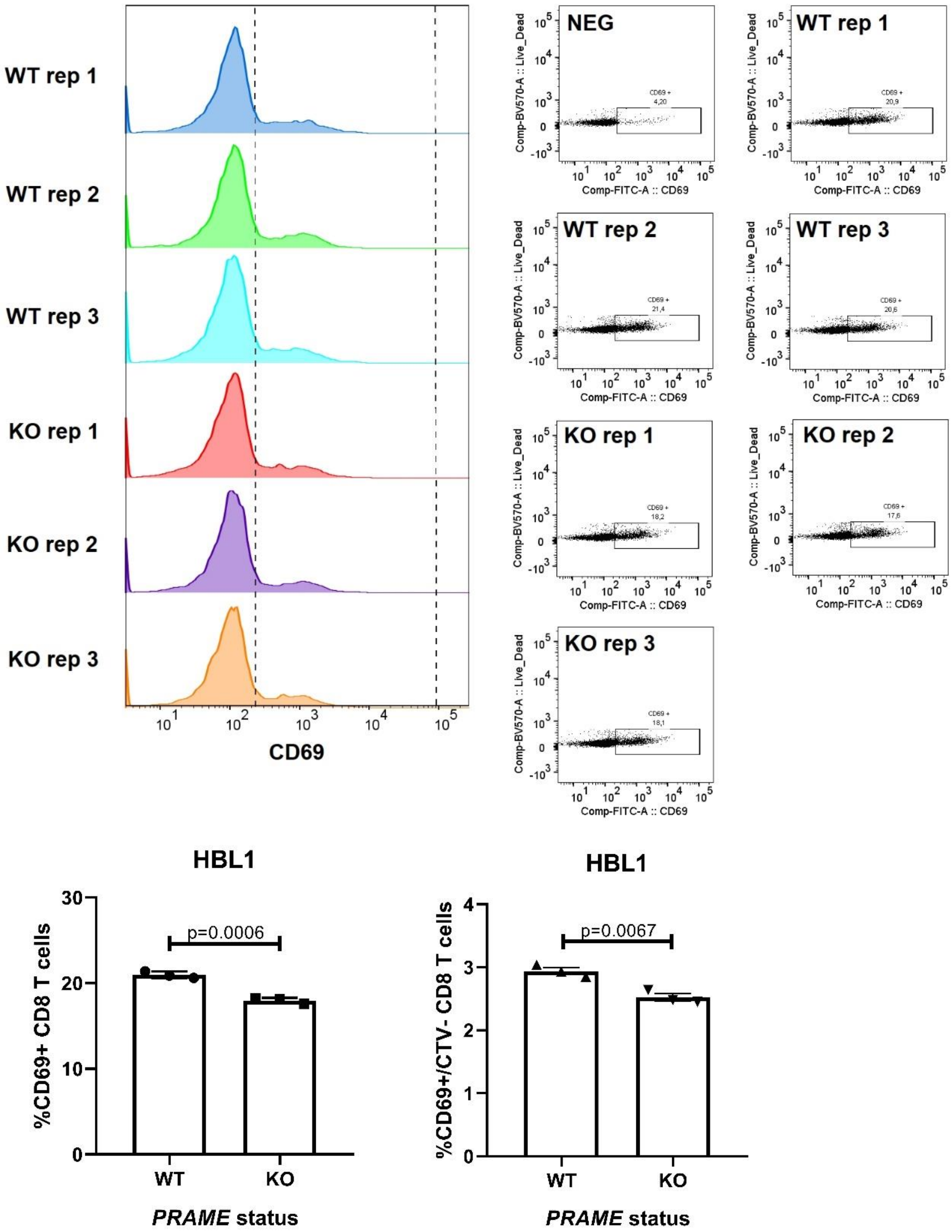

WT rep 1
WT rep 2
WT rep 3
KO rep 1
KO rep 2
KO rep 3
CD69
NEG
Comp-BV570-A :: Live_Dead
Comp-FITC-A :: CD69
CD69 +
4,20
20,9
21,4
20,6
18,2
17,6
18,1
HBL1
%CD69+ CD8 T cells
p=0.0006
%CD69+/CTV- CD8 T cells
p=0.0067
WT
KO
PRAME status

B

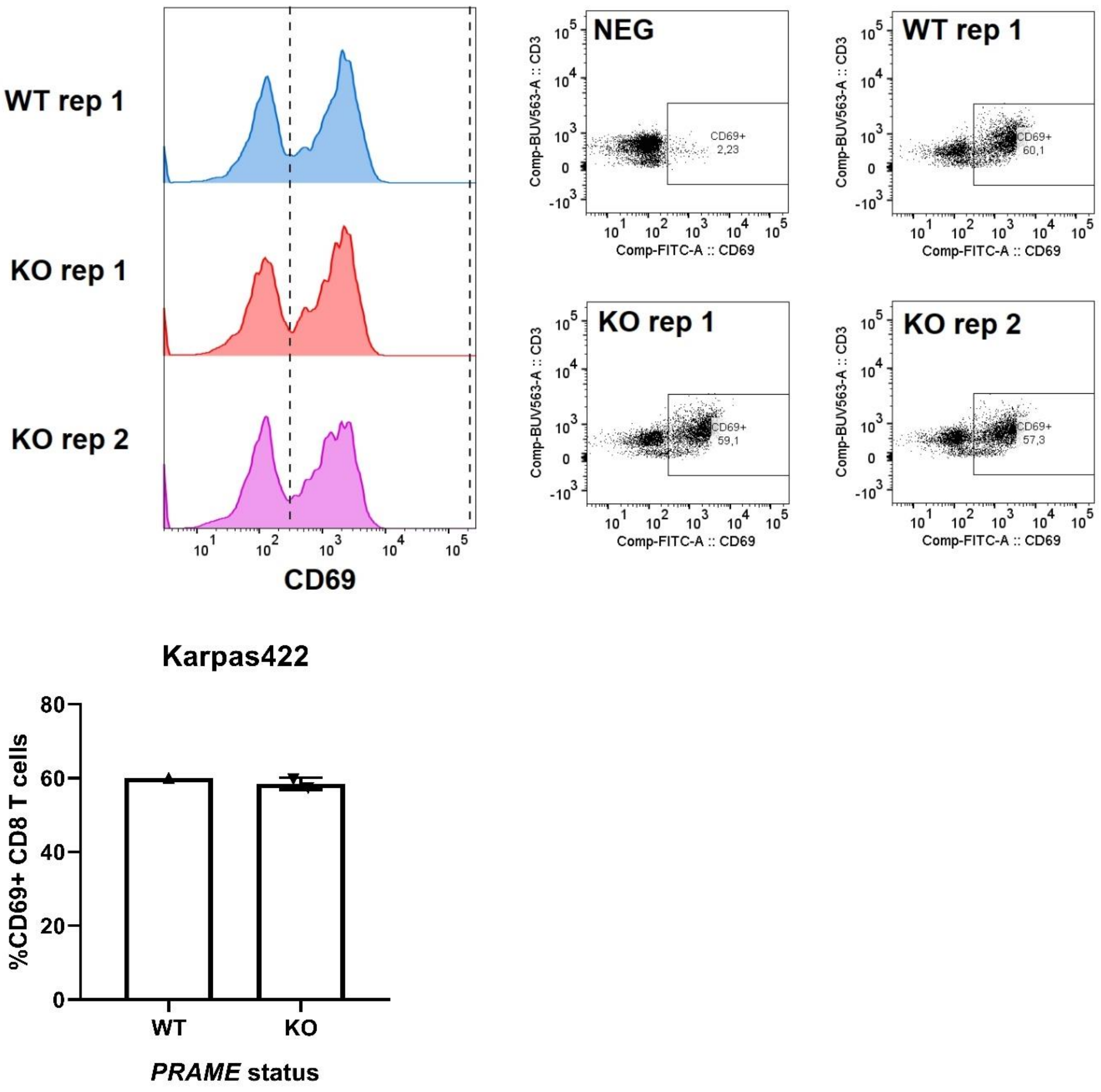

WT rep 1
KO rep 1
KO rep 2
CD69
NEG
WT rep 1
KO rep 1
KO rep 2
Comp-BUV563-A :: CD3
Comp-FITC-A :: CD69
CD69+
2,23
60,1
59,1
57,3
Karpas422
%CD69+ CD8 T cells
WT
KO
PRAME status

C

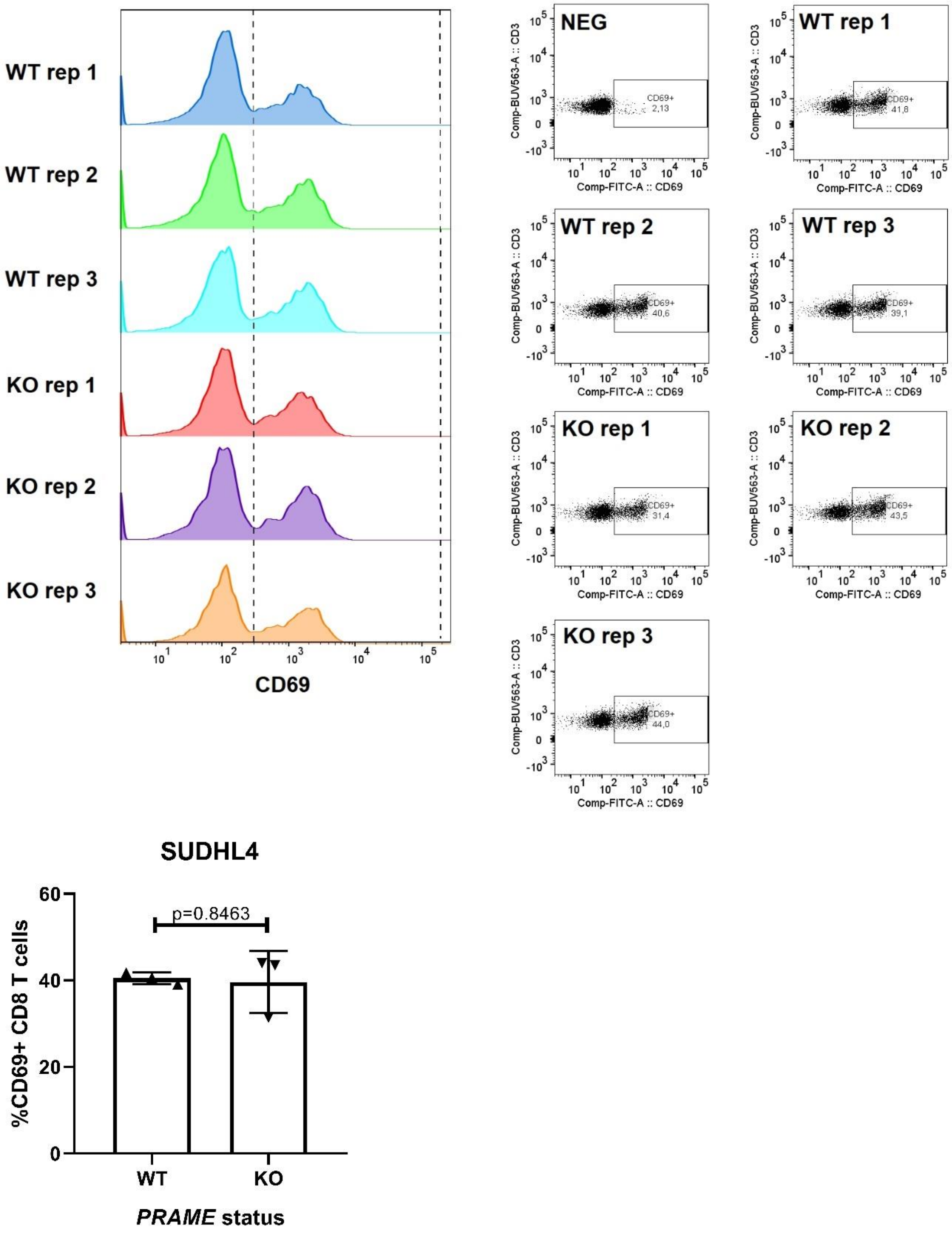

WT rep 1
WT rep 2
WT rep 3
KO rep 1
KO rep 2
KO rep 3
CD69
NEG
CD69+
2,13
41,8
40,6
39,1
31,4
43,5
44,0
Comp-BUV563-A :: CD3
Comp-FITC-A :: CD69
SUDHL4
p=0.8463
%CD69+ CD8 T cells
WT
KO
PRAME status

D

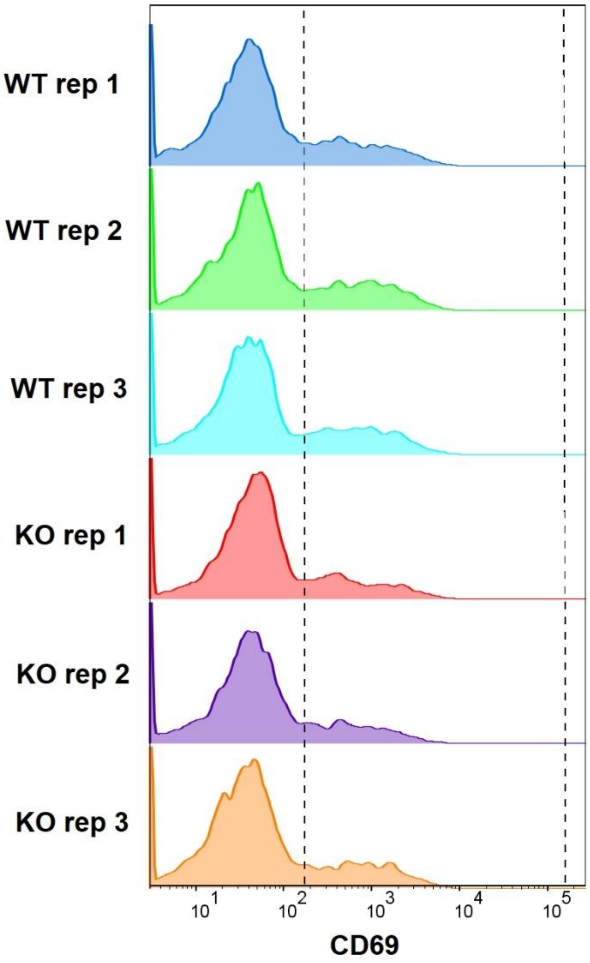

WT rep 1
WT rep 2
WT rep 3
KO rep 1
KO rep 2
KO rep 3
CD69

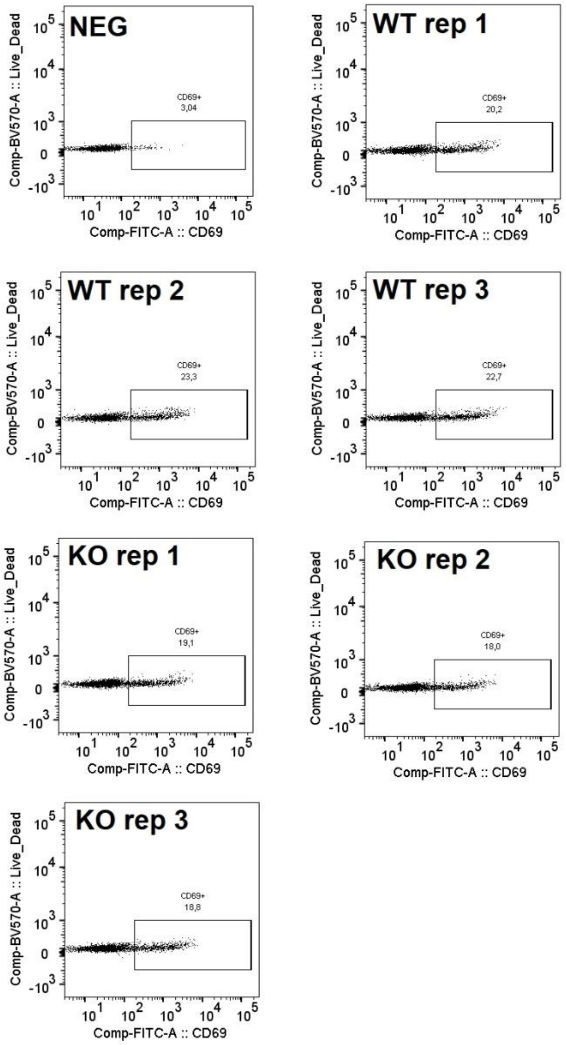

NEG
CD69+ 3,04
WT rep 1
CD69+ 20,2
WT rep 2
CD69+ 23,3
WT rep 3
CD69+ 22,7
KO rep 1
CD69+ 19,1
KO rep 2
CD69+ 18,0
KO rep 3
CD69+ 18,8
Comp-BV570-A :: Live_Dead
Comp-FITC-A :: CD69

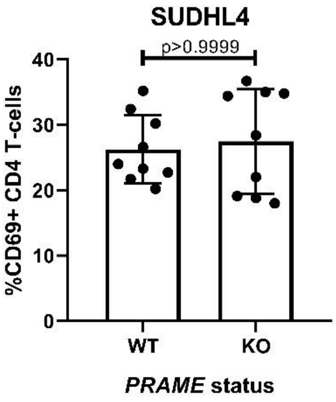

SUDHL4
p>0.9999
%CD69+ CD4 T-cells
WT
KO
PRAME status

Figure S6. Co-culture analysis on SU-DHL-4 and HBL-1 cells

(A) CD69+ CD8 T-cell populations (upper: FACS plot, lower left: bar-graph for CD69+ population, lower right: CD69+ CTV population) for co-cultured with HBL-1 PRAME isogenic cell lines.
(B) CD69+ CD8 T-cell populations (upper: FACS plot, lower: bar-graph) for co-culutured with Karpas-422 PRAME isogenic cell lines.
(C) CD69+ CD8 T-cell populations (upper: FACS plot, lower: bar-graph) for co-cultured with SU-DHL-4 PRAME isogenic cell lines.
(D) CD69+ CD4 T-cell populations (upper: FACS plot, lower: bar-graph) for co-cultured with SU-DHL-4 PRAME isogenic cell lines.

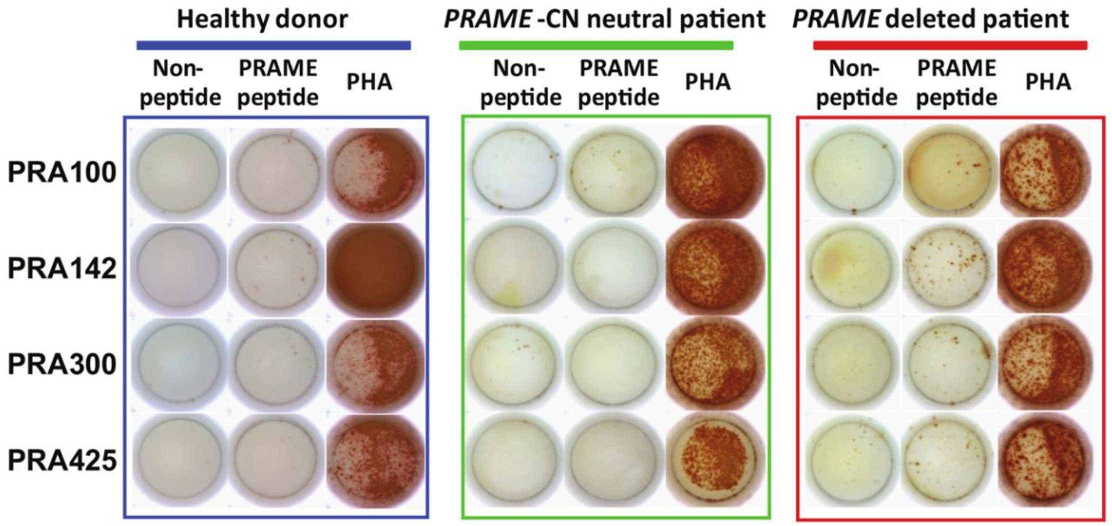

Figure. S7. Representative IFN γ ELISPOT assay results of healthy donor, PRAME CN-neutral, and *PRAME* deleted patient derived T-cells.

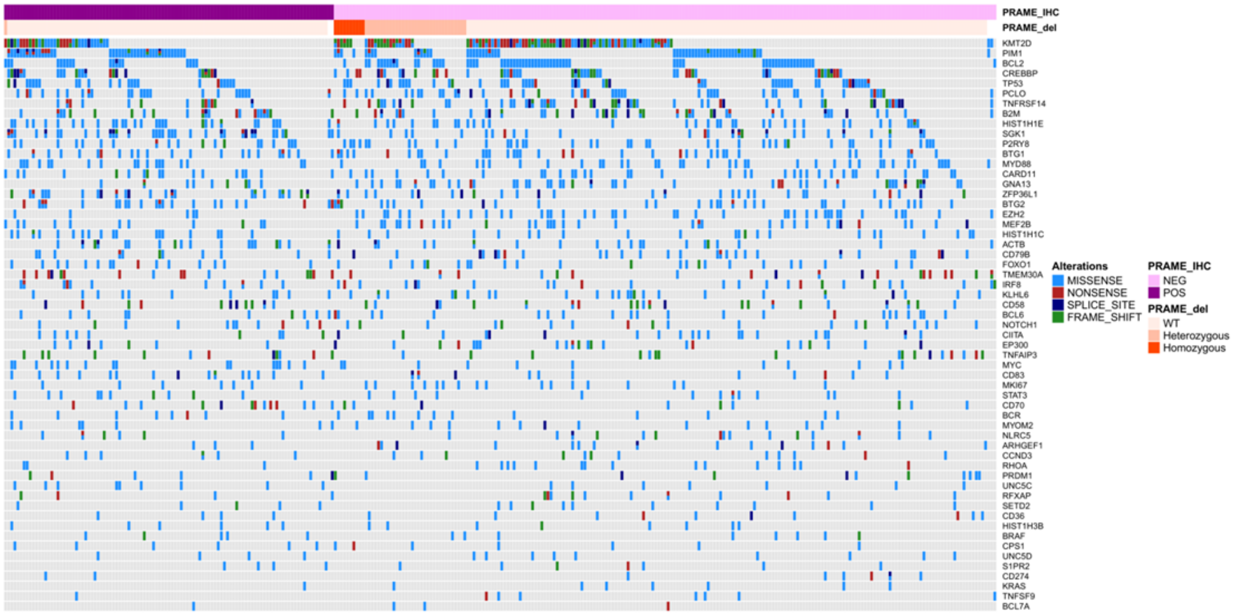

Figure.S8. Mutation oncoprint between PRAME IHC-negative and IHC-positive samples.

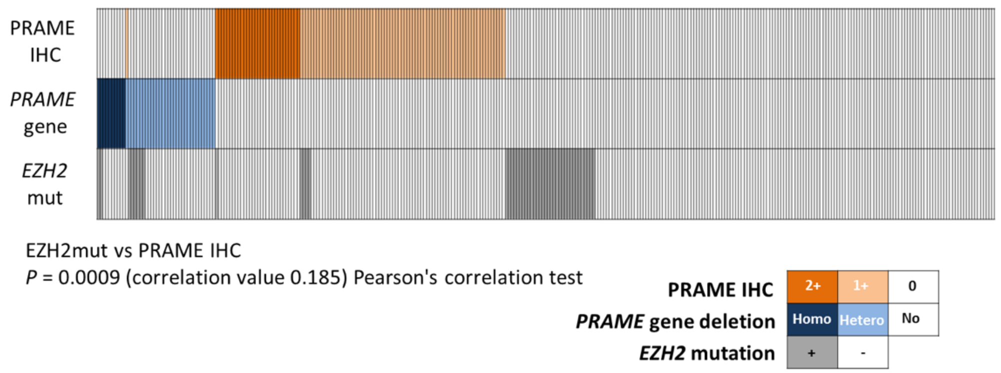

	PRAME-CN-neutral	PRAME-deleted
EZH2-mut	37	8
EZH2-wt	257	36

Fisher exact test: *P* = 0.3398

Figure S9. Correlation among PRAME-IHC status, *PRAME*-CN, and *EZH2*-mutation.

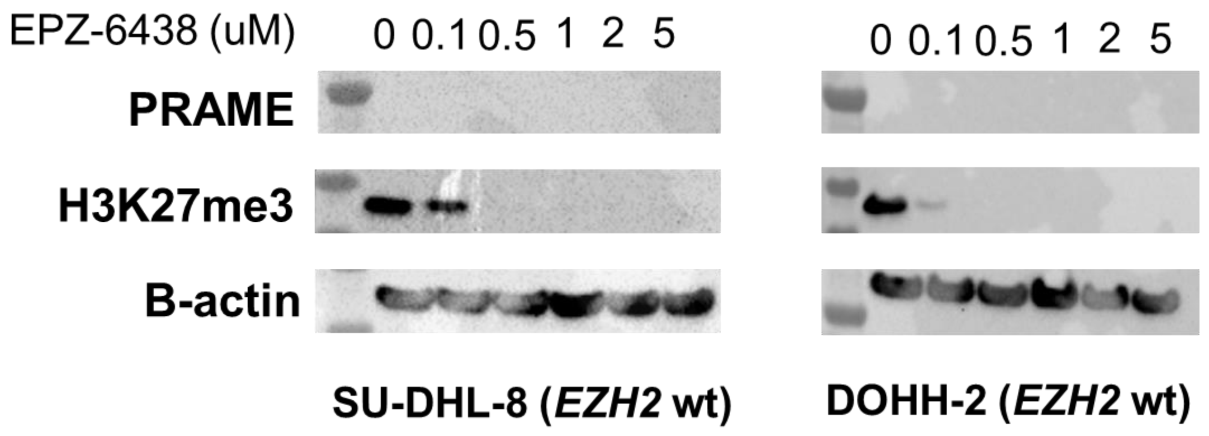

Figure. S10. Immunoblot for EPZ-6438 treated cells (*EZH2* wt).
Protein lysate (anti-PRAME and anti-H3K27me3: 20μg, anti-B-actin: 20μg) was loaded in the separate gels and transferred to separate membranes).

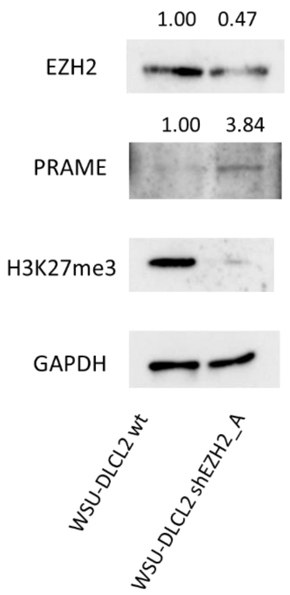

Figure. S11. EZH2, PRAME, H3K27me3 and GAPDH Immunoblot for EZH2 knock-down by shEZH2 system. Protein lysate (anti-EZH2, anti-PRAME, anti-H3K27me3: 20μg, anti-GAPDH: 5μg) was loaded in the separate gels and transferred to separate membranes. Detecting anti-PRAME membrane was visualized using high-sensitivity chemiluminescent reagent.

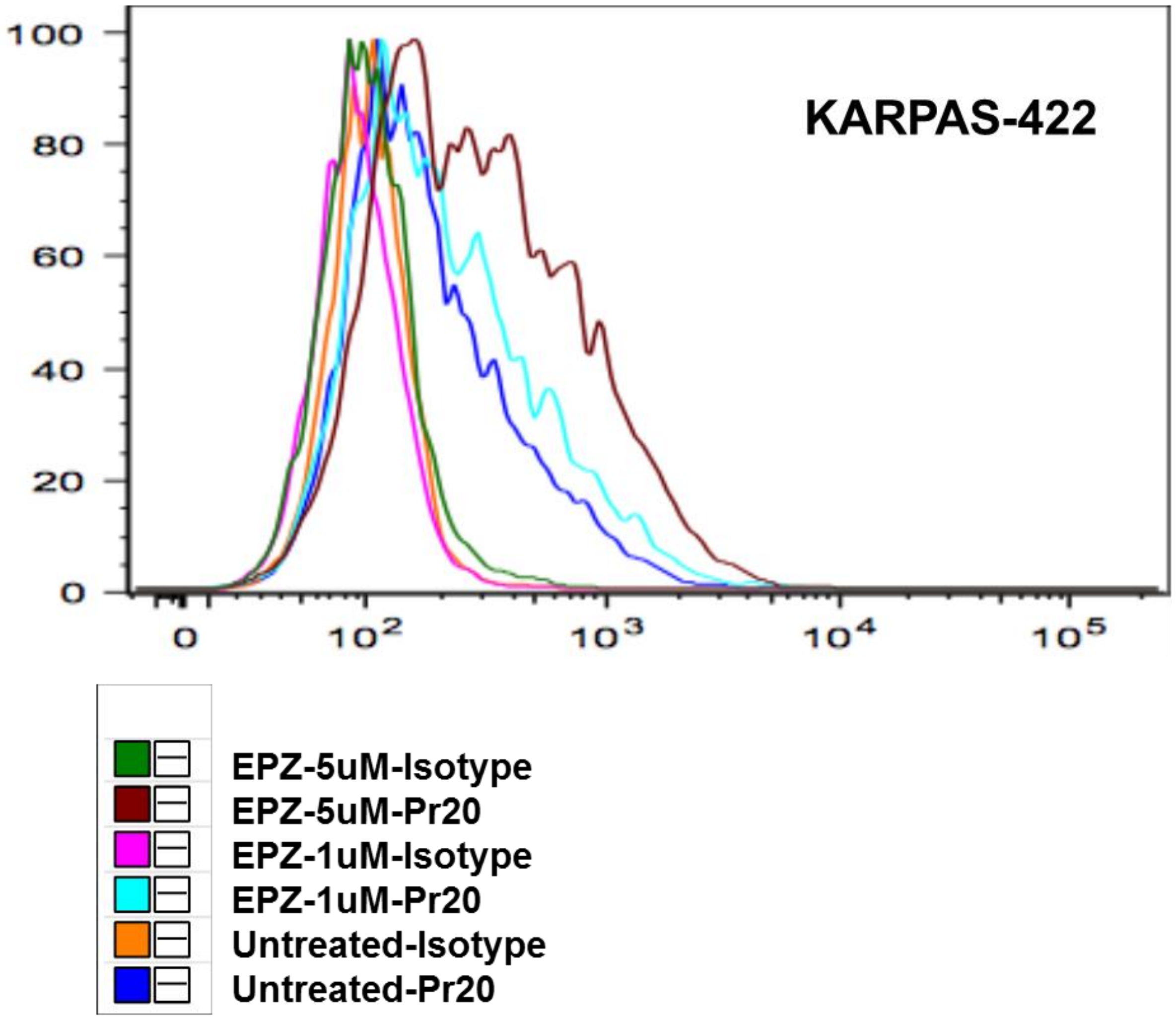

Figure.S12. Pr20 binding assay in DLBCL cell line.
Karpas-422 cell line was treated with EPZ-6438 at concentrations of 1uM and 5uM, for 4 days. The cells were harvested, washed and stained with Pr20 mAb or its isotype control hIgG1 conjugated to APC, at a concentration of 3 ug/ml. Data show one of two separate experiments. Both experiments showed binding of Pr20 to the cells, which was enhanced dose-dependently by treating cells with EPZ.

Karpas422
SU-DHL-4

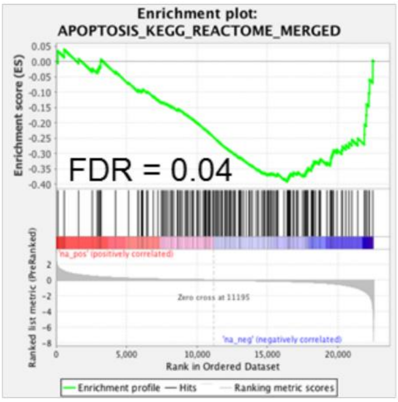

Figure. S13. Pre-ranked GSEA enrichment plots of apoptosis pathway genes in *PRAME* wt versus *PRAME* KO cell lines.

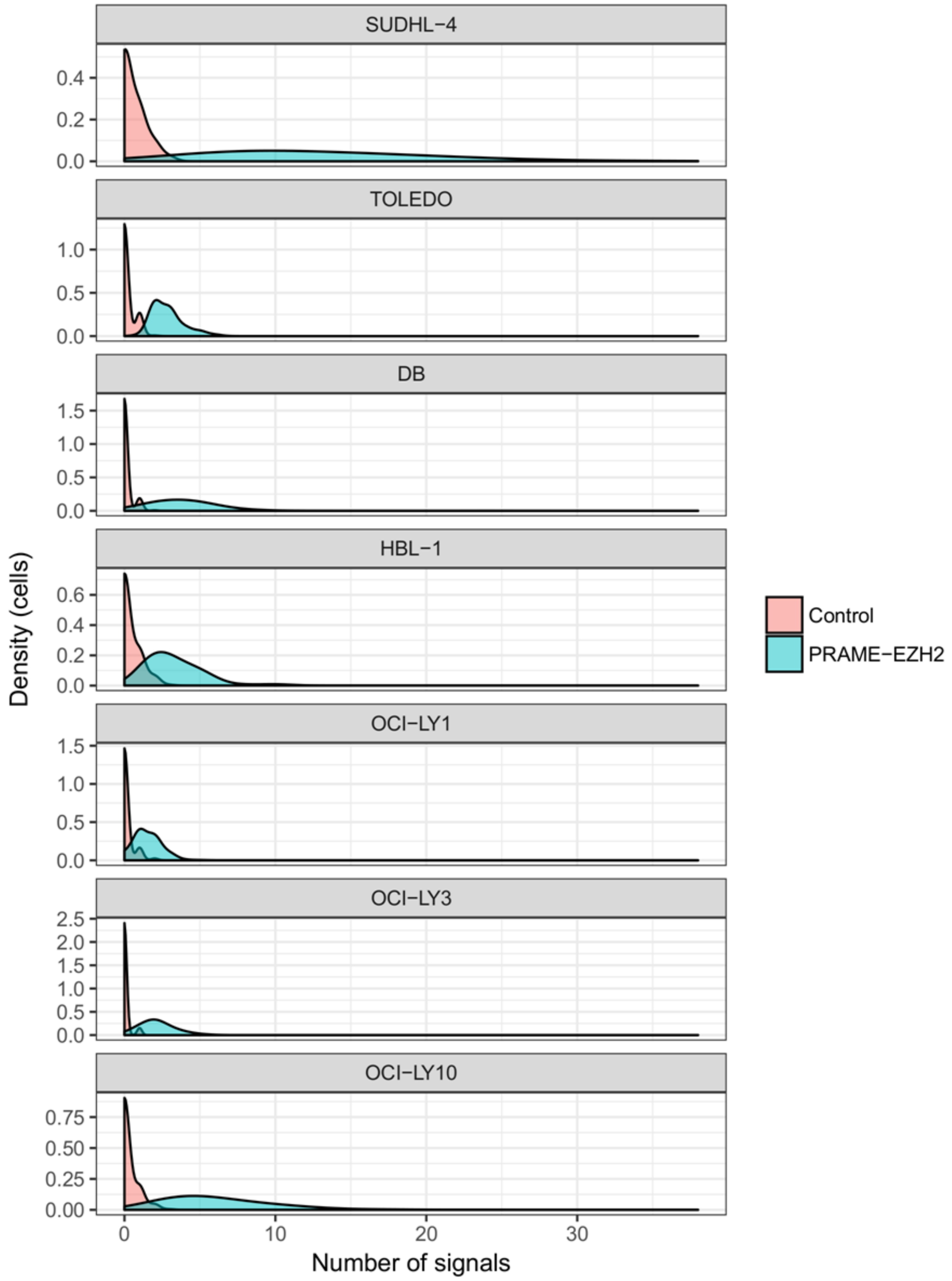

Figure.S14. PRAME-EZH2 PLA results in each cell line. Red peak shows control analysis (PRAME antibody/EZH2 antibody only) and blue peak shows EZH2-PRAME combined antibodies.

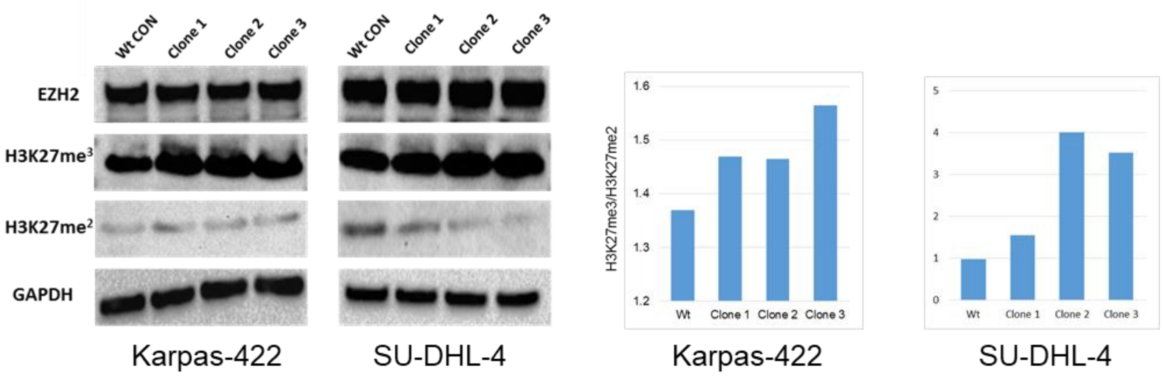

Figure.S15. EZH2/PRC2 activity change in PRAME isogenic KO cell lines. Immunoblotting of EZH2, H3K27me3, H3K27me2 in Karpas-422 and SU-DHL-4 isogenic PRAME KO cell lines (left). Densitometry of H3K27me3/H3K27me2 (right). Protein lysate (anti-EZH2, anti-H3K27me3, anti-H3K27me2: 20μg, anti-GAPDH: 5μg) was loaded into separate gels and transferred to separate membranes).
